# Alcoholic fatty liver disease inhibited the co-expression of Fmo5 and PPARα to activate the NF-κB signaling pathway, thereby reducing liver injury via inducing gut microbiota disturbance

**DOI:** 10.1186/s13046-020-01782-w

**Published:** 2021-01-07

**Authors:** Lingjian Kong, Jing Chen, Xiaoli Ji, Qian Qin, Huiyu Yang, Dan Liu, Deliang Li, Meiling Sun

**Affiliations:** 1 Department of Gastroenterology, The First Affiliated Hospital of Zhengzhou University, No.1 Jianshe East Road, Henan Province Zhengzhou, 450052 PR China; 2 Department of Gastroenterology, The Second Affiliated Hospital of Harbin Medical University, Harbin, Heilongjiang 150086 PR China; 3 Department of Intervention, The First Affiliated Hospital of Zhengzhou University, Zhengzhou, Henan Province 450052 PR China; 4 Physical Examination Center, The First Affiliated Hospital of Zhengzhou University, Zhengzhou, Henan Province 450052 PR China; 5 Department of Gastroenterology, ZhuJiang Hospital of Southern Medical University, Guangzhou, Guangdong Province 510280 PR China

**Keywords:** Alcoholic fatty liver disease, Fmo5, PPARα, NF-κB, Liver injury, Gut microbiota disturbance

## Abstract

**Background:**

Alcohol-induced intestinal dysbiosis disrupts and inflammatory responses are essential in the development of alcoholic fatty liver disease (AFLD). Here, we investigated the effects of Fmo5 on changes in enteric microbiome composition in a model of AFLD and dissected the pathogenic role of Fmo5 in AFLD-induced liver pathology.

**Methods:**

The expression profile data of GSE8006 and GSE40334 datasets were downloaded from the GEO database. The WGCNA approach allowed us to investigate the AFLD-correlated module. DEGs were used to perform KEGG pathway enrichment analyses. Four PPI networks were constructed using the STRING database and visualized using Cytoscape software. The Cytohubba plug-in was used to identify the hub genes. Western blot and immunohistochemistry assays were used to detect protein expression. ELISA assay was used to detect the levels of serum inflammatory cytokines. Lipid droplets in the cytoplasm were observed using Oil Red O staining. Apoptosis was detected using a TUNEL assay and flow cytometry analysis. ROS levels were detected using flow cytometry analysis. Nuclear translocation of NF-κB p65 was observed using immunofluorescence staining. Co-immunoprecipitation was used to detect the co-expression of PPARα and Fmo5 in L02 cells. 16S rDNA sequencing defined the bacterial communities in mice with AFLD.

**Results:**

Fmo5 is a key DEG and is closely associated with the gut microbiota and PPAR signaling pathway. Gut microbiome function in AFLD was significantly related to the PPAR signaling pathway. AFLD induced shifts in various bacterial phyla in the cecum, including a reduction in *Bacteroidetes* and increased *Firmicutes*. Fmo5 and PPARα co-expression in cell and animal models with AFLD, which decreased significantly. Silencing of Fmo5 and PPARα aggravated the functions of AFLD inducing apoptosis and inflammatory response, promoting liver injury, and activating the NF-κB signaling pathway in vivo and in vitro. The NF-κB inhibitor abolished the functions of silencing of Fmo5 and PPARα promoting AFLD-induced apoptosis, inflammatory response, and liver injury.

**Conclusion:**

Our data indicated that the co-expression of Fmo5 and PPARα was involved in AFLD-related gut microbiota composition and alleviated AFLD-induced liver injury, apoptosis, and inflammatory response by inhibiting the nuclear translocation of NF-κB p65 to inhibit the NF-κB signaling pathway.

**Supplementary Information:**

The online version contains supplementary material available at 10.1186/s13046-020-01782-w.

## Background

The toxic effect of alcohol can impact the important organs of the body, especially the liver [1]. Because of the prevalence of wine culture, the incidence of alcoholic liver disease is increasing annually in China. When the body is stimulated by alcohol, the excessive input of lipids leads to the disturbance of lipoprotein synthesis and metabolism in the liver, and the insufficient oxidation of fatty acids, which leads to the deposition of fat in the liver, resulting in fatty liver. Alcoholic fatty liver disease (AFLD) is caused by long-term heavy drinking and is the most common and reversible stage of alcoholic liver disease [1–4]. Although numerous studies have been conducted on the pathogenesis of alcoholic fatty liver at home and abroad [3], there is still no effective treatment for alcoholic fatty liver in the clinic. Therefore, a systematic study of the pathogenesis of AFLD can provide a more effective treatment for alcoholic liver disease.

The intestinal flora is a diverse microecosystem composed of more than 2000 species of bacteria and has 150 times more genomes than the human genome [5]. Intestinal microflora can affect the basic functions of the host, such as digestion and absorption, energy metabolism and immune defense by regulating endocrine, nerve, and immunity functions [6, 7]. The composition of intestinal microflora is affected by the external environment, diet and host itself. The imbalance of intestinal flora can cause the body to be in an extended state of chronic inflammation for a long time [8]. Alcoholic fatty liver is closely related to the changes in intestinal flora [9, 10]. Therefore, to explore the specific mechanism of the inflammatory response induced by intestinal flora imbalance in AFLD needs to be further studied.

Flavin-containing monooxygenases (FMOs) catalyze the ingredients of medicine and diet and have a unique catalytic mechanism [11, 12]. Flavin-containing monooxygenase 5 (Fmo5) is a member of the FMOs family, and its expression in the liver tissue of aging mice is lower than that in normal mice [13]. *Salvia miltiorrhiza* shows significantly upregulated expression in the injured liver [14]. FMO5 is a regulator of glucose homeostasis and a potential sensor for gut bacteria [15]. This shows that Fmo5 has a protective effect on the liver and is closely related to the balance of intestinal flora. Our studies have shown that Fmo5 is significantly associated with intestinal flora and alcoholic diet. Therefore, the role of Fmo5 in AFLD-mediated intestinal flora disorder and liver injury needs to be further studied.

## Methods

### Data sources

The NCBI-GEO database is a free and public database containing gene profiles. Two microarray datasets (GSE8006 about intestinal tract and GSE40334 about liver) were obtained from the GEO database (https://www.ncbi.nlm.nih.gov/gds/). The gene expression profiles of the GSE8006 dataset included 4 control liquid diet group samples and 4 alcohol liquid diet group samples. The gene expression profiles of the GSE40334 dataset included 3 colon germ free group samples and 3 colon specific pathogen-free group samples.

### Identification of DEGs

GEO2R is an interactive web tool that can compare and analyze two different groups of samples under the same experimental conditions. In this study, DEGs in the GSE8006 dataset and GSE40334 dataset were analyzed using GEO2R. Subsequently, DEGs met the cutoff criteria of the adjusted *P-value* < 0.05 and |log fold change (FC)| > 1.0.

### Functional enrichment analysis of DEGs

GO functional analysis and KEGG pathway analysis were perform to predict the potential functions and the main signaling pathways involved of the DEGs using the Database for Annotation, Visualization and Integrated Discovery (DAVID; https://david.ncifcrf.gov/; version 6.8) database. DEGs of GSE8006 and GSE40334 datasets were submitted to the DAVID online program. The top 10 items of the cellular component (CC), biological process (BP), and molecular function (MF) categories and KEGG pathways were then sorted and presented in the form of bubble maps. These bubble plots were drawn using the ggplot2 R package based on *P-value* (*< 0.05* was considered statistically significant) through the statistical software R (version 3.6.1).

### PPI network construction and hub gene identification

The Search Tool for the Retrieval of Interacting Genes database (Version 10.0, http://string-db.org) database was used to predict potential interactions between gene candidates at the protein level. A combined score of > 0.4 (medium confidence score) was considered significant. Additionally, cytoscape software (Version 7.2.0, http://www.cytoscape.org/) was utilized for constructing a PPI network. CytoHubba, a Cytoscape plugin of identifying hub objects and sub-networks from complex interactome, was utilized to explore PPI network hub genes; it provides a user-friendly interface to explore important nodes in biological networks and computes using eleven methods, of which MCC has a better performance in the PPI network.

### Weighted gene co-expression network analysis (WGCNA)

The WGCNA R package was used to construct a weighted correlation network between the prognostic genes. In the network, we used the pairwise Pearson coefficient to evaluate the weighted co-expression relationship between all genes in the adjacency matrix. The soft threshold was used to ensure a scale-free network. In the unsigned co-expression network, genes with high absolute correlations were clustered into the same module. The modules were also identified by hierarchical clustering of the weighted coefficient adjacency matrix to calculate the topological overlap matrix (TOM). The absolute value of the threshold of correlation degree > 0.8. In addition, the topological overlap of the intramodules and adjacency modules was used to select the functional modules.

### 16S rDNA sequencing

Cecal contents were collected from some animals and frozen at − 80 °C. DNA was extracted using Stool DNA Extraction Kit (Qiagen) according to the manufacturer’s instructions. To check DNA quality and 16S content prior to sequencing, universal primers were used for SYBR Green quantitative polymerase chain reaction (qPCR) with the following extended cycling protocol: 95 °C 10 min; 95 °C 15 s, 60 °C 30 s, 72 °C 30 s for 40 cycles. Sequencing was completed at the Cincinnati University Children’s Hospital Medical Center’s DNA Sequencing and Genotyping Facility Core (Cincinnati, OH) as described [14]. All antibiotic-treated samples failed to yield 16S rDNA sequence data; one sample each from the ethanol- and pair-fed groups was excluded based on insufficient sequence data.

UPARSE and UTAX (http://www.drive5.com/usearch/manual/cmd_utax.html) were used to generate OTU tables from 16S rDNA read data and to make taxonomic assignments. QIIME package scripts were used for calculations of α- (*PD_whole_tree*, *chao1*, *observed_otus* and *shannon*) and β- (*Bray-Curtis*, *Un-Weighted UniFrac*, and *Weighted UniFrac*) diversity.

### Animal experimentation

Male C57BL/6 J mice (22 ± 2 g, 6 weeks old) and Fmo5 gene knockout (Fmo5^−/−^) mice (20 ± 3 g, 6 weeks old) were purchased from the Beijing Vital River Laboratory Animal Technology Co., Ltd. (Beijing, China) and housed individually in cages with a 12-h light/dark cycle at 23 ± 2 °C with full access to chow diet and water. All experimental protocols were approved by the Institutional Animal Care and Use Committee of the Zhengzhou University.

The AFLD mouse model was established using a modified Lieber–DeCarli liquid diet (21). The 40 mice were separated into four groups, each consisting of 10 mice. The first group (control group) and the third group (Fmo5^−/−^ group) were fed with a normal standard growth diet while the other two groups (AFLD group and Fmo5^−/−^_AFLD group) were fed high-fat liquid diets (35% fat, 18% protein, and 47% carbohydrates, provided by TROPHIC Animal Feed High-Tech Co., Ltd., Nantong, China) for an acclimation period of 2 weeks acclimation. The amount of ethanol included increased over 2 weeks to reach a final concentration of 20% (*v*/*v*). Body weight gain and food intake were assessed once a week. The pair-fed control group (Con) was included in this model.

After 12 weeks, the fasted mice were euthanized, and blood samples were collected. The feces in the colon were also harvested into 2-ml sterile tubes to assess the gut microbiota. The tissues were immediately removed and weighed, and the liver coefficient (liver weight/body weight) was calculated. The left lobe of the liver was instantly fixed in 10% buffered formalin for histological analysis with the rest of the tissues frozen in liquid nitrogen and stored at − 80 °C until further use.

### Hepatic triglyceride staining

The liver tissue was separated and rapidly fixed in 4% neutral buffered formalin solution for 24 h and then processed for paraffin embedding. Five-micrometer-thick paraffin sections were stained with hematoxylin and eosin (H&E) and oil-red solution. The liver steatosis status was examined under a light microscope (Olympus, Tokyo, Japan), and photographed at 100 × and 200 × magnification.

### Measurement of liver function and serum pro-inflammatory cytokines

Blood samples were obtained from the common carotid artery to detect serum alanine aminotransferase (ALT), aspartate aminotransferase (AST), tumor necrosis factor-α (TNF-α) and interleukin-6/− 4 (IL-6/− 4) when rats were sacrificed at 24 h after reperfusion. The levels of serum ALT and AST were measured using corresponding kits (Jiancheng, Nanjing, China) to assess the liver function. Serum TNF-α, IL-6, and IL-4 levels were measured by radioimmunoassay kits (Albert Poole Biotechnology, Beijing, China).

### Apoptosis and immunohistochemical examination of liver

Harvested livers were placed into 10% formalin immediately after excision, and immersed for 24 h. Liver specimens were then embedded in paraffin, and sections were cut at 5 μm. Sections were stained for: (1) Apoptosis was detected by the TdT-mediated dUTP-biotin nick end labeling (TUNEL) method using an in situ apoptosis detection kit (Boster Biological Engineering, Wuhan, China) as previously described. The number of cells with TUNEL-positive nuclei was counted from 20 randomly selected fields at × 100 or × 200 magnification per liver sample. Results were expressed as the mean number of TUNEL-positive apoptotic hepatocytes per microscopic field; and (2) Immunobiological analysis of Fmo5, caspase-3, NF-κB p65 expression in paraffin wax-embedded liver sections were performed using a standard peroxidase-antiperoxidase technique as described previously, using a Fmo5 rabbit anti-mouse polyclonal antibody (Abcam Inc., United Kingdom) or mouse monoclonal antibodies against caspase-3 and NF-κB p65 (Santa Cruz, CA, United States) at a 1:150 dilution, with a biotinylated goat anti-rabbit or goat anti-mouse antibody (Santa Cruz, CA, United States) as the secondary antibody. Brown color in the cytoplasm of the hepatocytes was evaluated as positive staining.

### Cell culture

Human normal hepatocyte cell line L02 was purchased from the Chinese Academy of Science (Shanghai, China). Cells were cultivated in RPMI-1640 medium (HyClone, Logan, UT, USA) supplemented with 10% heat-inactivated fetal bovine serum (FBS), 100 U/ml penicillin, and 100 μg/ml streptomycin according to the manufacturer’s instructions. Cells were maintained at 37 °C in a humidified atmosphere with 5% CO_2_. Half of the growth medium was changed each day. L02 cells at 60–70% confluency after the fourth passage were plated for the experiments.

### Construction of AFLD cell model

To deplete L02 cells of iron, the chelator, desferrioxamine mesylate (DFO) (100 μM, D9533; Sigma-Aldrich), was added to the cell culture medium [16]. Iron loading of L02 cells was achieved by incubating cells in the presence of iron-dextran (0–100 μg/ml; Sigma-Aldrich). Cells were collected 24 h after treatment and at 48 h after treatment for extraction of mRNA and protein, respectively. For alcohol treatment, L02 cells were treated for 48 h with 200 nM alcohol (Sigma-Aldrich); then, cells were treated for 24 h with DFO (100 μM) in the presence of 200 mM alcohol.

### Cell viability

The cells were seeded in 96-well plates at a density of 5 × 10^3^ cells/well and cultured overnight. Subsequently, the cells were pretreated with various concentrations of ethyl alcohol (0–200 nM) for 72 h at 37 °C. Cell viability was estimated using the cell counting kit-8 colorimetric assay (CCK-8 assay, Dojindo Molecular Technologies, Inc., Kumamoto, Japan). In addition, the viability was estimated using the cell counting kit-8 colorimetric assay.

### Oil red O staining

The Oil Red O stock solution was composed of 5 g Oil Red O in 100 ml isopropyl alcohol. Fresh Oil Red O working solution was prepared by diluting the Oil Red O stock with distilled water in a 3:2 ratio. Cells were washed twice in PBS after PA treatment, fixed with 4% formaldehyde for 30 min, and stained with Oil Red O working solution for 40 min at room temperature. Then, L02 cells were washed with 60% isopropyl alcohol once followed by washing with water twice. The nuclei of the cells were stained with Hematoxylin. The number of Oil Red O-positive cells was counted in replicates in 15 randomly selected fields at × 100 and × 200 magnification under a bright-field microscope.

Double immunofluorescent staining.

The L02 cells were simultaneously incubated with primary antibodies against Fmo5 (1:50) and PPARα (1:50). The effect concentration of tetraethyl rhodamine isothiocyanate (TRITC) and fluorescein isothiocyanate (FITC) was 1:50. 4′,6-diamidino-2-phenylindole (DAPI) counterstained nuclei. At least three independent experiments were done.

### Co-immunoprecipitation (co-IP)

For Co-IP, anti-Fmo5 was coupled with protein A-Sepharose beads (Sigma) in RIPA buffer overnight at 4 °C. The immune complex was then added to cell lysate and incubated at 4 °C for 2 h. Endogenous Fmo5 was immunoprecipitated using an Fmo5 antibody (Santa Cruz Biotechnology, Inc., Dallas, TX, USA). The IP samples were analyzed by western blot using the following antibodies: Fmo5 (Abcam, Cambridge, MA, USA) and PPARα (Abcam).

### Triglyceride (TG) assay

Cells from the different groups were harvested and washed twice with PBS. The intracellular triglycerides were measured using a triglyceride assay kit according to the manufacturer’s instruction (Applygen Technologies Inc., Beijing, China). The TG concentrations were normalized to the total cell protein concentration.

### Intracellular ROS measurement

The intracellular ROS level is measured using the probe 2′,7′-dichlorofluorescein diacetate (DCFH-DA; Solarbio; Beijing; China). Briefly, cells were seeded in a 96-well plate at a density of 2 × 104 cells/well and cultured with their respective treatments for 24 h. After incubation with 10 μM DCFH-DA for 30 min at 37 °C, cells were washed twice with PBS. The fluorescence intensity is monitored using an excitation wavelength of 488 nm and an emission wavelength of 530 nm using a fluorescence microplate reader (Molecular Devices, Sunnyvale, CA).

### Cell transfection

SiRNA was used to specifically target Fmo5 or PPARα. L02 cells were transiently transfected with Fmo5 siRNA or PPARα siRNA (Genesis Biotechnology, Wuhan, China) or negative control siRNA (Santa Cruz, CA, USA) according to the standard protocols. Briefly, cells were seeded at 2 × 10^5^ in 12-well plates without antibiotics. Next, cells with 30–50% confluence were transfected with 50 nM siRNA or negative control using Lipofectamine 2000 (Invitrogen, USA) according to the manufacturer’s protocol.

Fmo5 expressing plasmids or PPARα expressing plasmids were obtained from Genesis Biotechnology (Wuhan, China). Briefly, cells were seeded at 2 × 10^5^ in 12-well plates without antibiotics. Next, cells with 30–50% confluence were transfected with 30 nM expressing plasmids or negative control using Lipofectamine 2000 (Invitrogen, USA) according to the manufacturer’s protocol.

### Cell treatment

The cells were seeded in 96-well plates at a density of 5 × 10^3^ cells/well and cultured overnight. Subsequently, the cells were pretreated with different concentrations lipopolysaccharide (LPS; Sigma-Aldrich; 0, 50, and 100 ng/mL) for 12 h at 37 °C.

### Determination of apoptosis by Annexin V-FITC/PI staining

Chondrocyte apoptosis was determined by Annexin V-FITC/PI double labeling according to the manufacturer’s protocol. Briefly, after indicated cultures, chondrocytes were digested and suspended in binding buffer. Then 5 μl Annexin V and 5 μl PI solutions were added into cells and incubated for 15 min. Apoptotic rate was detected by BD FACSVerse™ flow cytometer (Becton Dickinson, Heidelberg, Germany) and analyzed by Cell Quest software (BD Biosciences). Experiments were conducted in triplicate.

### Intracellular reactive oxygen species (ROS) levels detection by flow cytometry

Intracellular reactive oxygen species (ROS) levels were measured by ROS Assay Kit (Beyotime, Shanghai, China) with an oxidation-sensitive fluorescent probe dye, DCFH-DA (2, 7- dichlorodihydro fluorescein diacetate). In brief, cells were seeded in 6-well plates (5 × 10^3^ cells per well) and incubated at 37 °C, 5% CO_2_ atmosphere for 24 h. Next, the cells were collected and washed with PBS, and then incubated at 37 °C with DMEM containing 10 μM DCFH-DA for 30 min. After that, cells were washed three times using serum-free DMEM. The samples were finally analyzed using a FACScan flow cytometer and Flowjo 7.6.1 software.

### Immunofluorescence staining

The cells were seeded onto 96 black well plate (5 × 10^4^ cells/well) and cultured until 80% confluence. After 30 min of incubation, cells were fixed with 4% paraformaldehyde and permeabilized with 0.05% Triton X. Then, cells were incubated overnight with primary antibodies against NF-ĸB p65 (1:400) at 4 °C. Samples were then washed with PBS, incubated for an hour with Alexa Fluor 488-conjugated goat anti-rabbit IgG (1:400), followed by DNA staining for 15 min with hoechst 33342 staining (1: 1000). The fluorescently labeled NF-ĸB p65 was detected by Operetta High-Content Imaging System.

### Western blot analyses of tissue and cells

Dissected livers were snap-frozen in liquid nitrogen and protein lysates for the detection of HIF1α and HIF2α proteins were extracted with the NE-PER kit (Pierce). Primary hepatocytes and Hep3B cells were lysed in buffer of 50 mM Тris–HCl, pH 8, 150 mM NaCl, 0.5% NP-40, 1 mM PMSF and a complete protease inhibitor cocktail tablet (Roche), kept on ice for 20 min and centrifuged at 12000 g for 20 min at 4 °C. 100 μg of proteins from liver or cells were resolved by SDS-PAGE, blotted, and probed with the following primary antibodies: Fmo5, PPARα, caspase-3, NF-kB p65, IL-6, IL-4, TNF-a, and GAPDH. The secondary antibody, either anti-mouse or anti-rabbit, was conjugated to horseradish peroxidase (1:5000; Bio-Rad Laboratories). The Pierce ECL system (ThermoScientific) was used for detection.

### RNA extraction and real-time PCR

The total RNA was extracted by using a TRIzol reagent. The first-strand cDNA was synthesized from 1 μg of total RNA using the Reverse Transcription System Bestar qPCR RT Kit according to the manufacturer instruction. Real-time PCR was carried out with an ABI 7500 Real-Time PCR System (Applied Biosystems, Lincoln Centre Drive, Foster City, CA 94404, USA). Each assay was performed in triplicate. The relative amount of Fmo5 and PPARα were calculated using with a 2^−ΔΔCt^ method and normalized using GAPDH as an internal control. The primers used in this study were shown below: for Fmo5, 5′-CCAGTTACGTGAATGATTCG-3′ (forward), 5′- AGCGCGTGTGAATGCAGGCC-3′ (reverse); for PPARα: 5′- CTTATAACTGCGGGAGGAC-3′ (forward), 5′-TACGTGCTGAACATGAT-3′ (reverse); for IL-6, 5′-CTGGTGTGTCACTGTCAACA-3′ (forward), 5′- CTGGAAACACGTGACAAAC-3′ (reverse); for IL-4, 5′-ACCTGTGTCGCTGAAACTGGGCCC-3′ (forward), 5′- TGGCGCAACACAGTGCACCA-3′ (reverse); for TNFα, 5′-CTGAACGCTGTGCTGAAAAGCT-3′ (forward), 5′- ATTCGCATTGTCATGCCATGTT-3′ (reverse); and for GAPDH: 5′-CATCGATTAGGGCATGCGC-3′ (forward), 5′- TCGTAACTAGGGCTACCGC-3′ (reverse).

### Measurement of TG content in liver

TG content in liver was determined as previously described. Briefly, 250 mg of liver sample was homogenized in 1.5 ml of methanol, and then added with 5.0 ml of MTBE and shaken at room temperature for 1 h. Subsequently, 1.25 ml of high purity water was added and mixed for 10 min, followed by centrifuging at 1000 g for 10 min, and the upper organic layer was collected. The aqueous layer was re-extracted with MTBE/methanol/water mixture (10/3/2.5 v/v/v; 2 ml) and the combined organic layers were dried under nitrogen. The extracted dried lipids were dissolved in a mixture of triton X-114/methanol (2:1 v/v, 60 μl) and analyzed for triglyceride (L-Type TGH) using commercially available kits (Wako Diagnostics, Richmond, VA, USA).

### Statistical analysis

Sequence data were processed using Quantitative Insights into Microbial Ecology (QIIME, version 1.9.1). The low-quality sequences, which had lengths of < 150 bp and average Phred scores of < 20 and contained ambiguous bases and mononucleotide repeats of > 8 bp, were filtered using the following criteria. Paired-end reads were assembled using FLASH. The remaining high-quality sequences were clustered into operational taxonomic units (OTUs) at 97% sequence identity by UCLUST (Edgar 2010). OTU taxonomic classification was conducted by BLAST and the OTUs containing more than 99.999% of the total sequences across all samples were reserved.

QIIME (version 1.9.1) was used to calculate all diversity indexes in these samples, and R software (version 3.5.2) was used for visual analysis. LEfSe software (LEfSe 1.0) was used for linear discriminant analysis. Principal coordinate analysis (PCoA) was performed using Bray-Curtis. The functions of gut microbiota were predicted for the CON and AFLD groups using Tax4Fun in the KEGG database.

All results are presented as means ± standard deviation. Data were analyzed with SPSS 19.0 using One-way analysis of variance (ANOVA), and, when appropriate, using a two-tailed Student’s *t* test between different groups. Differences among groups were evaluated for significance with the comparable variances, followed by Tukey’s and least significant difference (LSD) tests. *P* < 0.05 was considered statistically significant.

## Results

### AFLD regulates the gut microbiota composition in mice

**Fig. 1:**
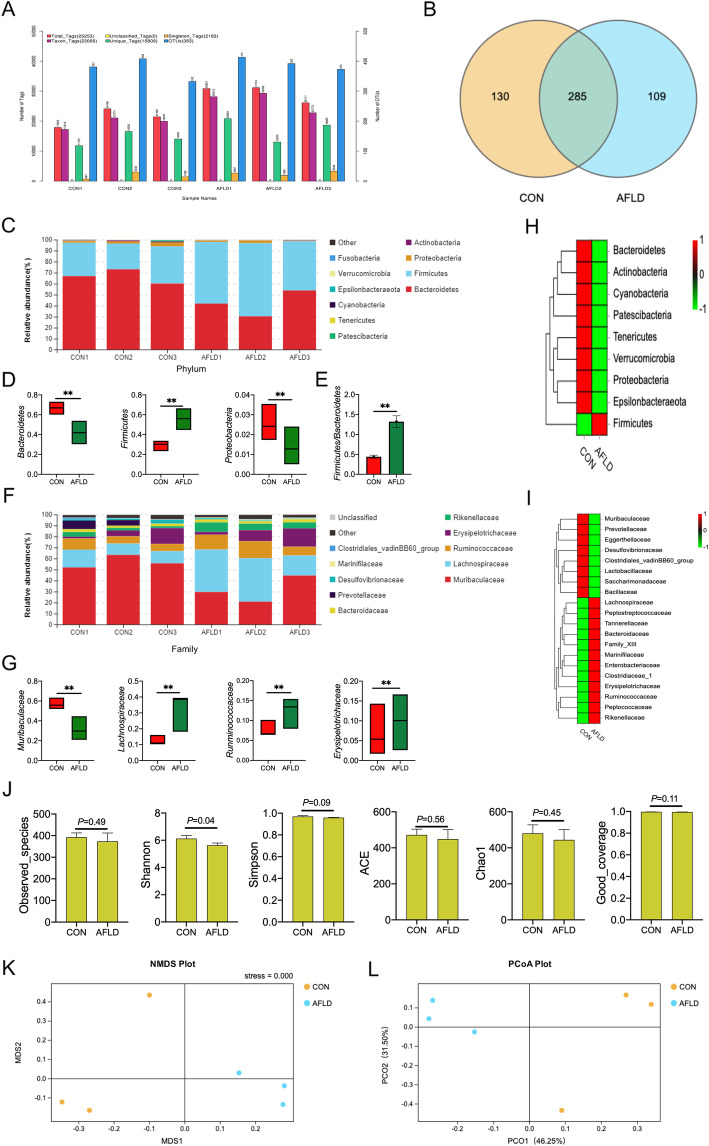
Bacterial community analysis and comparison. **a** Number of Tags/or 16S OTUs amongst mice with AFLD. **b** Core bacterial operational taxonomic units (OTUs) in mice from different groups. The core community was determined based on OTUs detected in every sample. The OTUs were assigned at a 97% sequences similarity threshold, and a Venn diagram was used to summarize the number of common OTUs between the two groups. The composition and abundance distributions of each group at the phylum (**c**) and family (**f**) levels were shown using QIIME software. At the phylum (**d** and **e**) and family (**g**) levels, pairwise comparisons, conducted to determine the sequence amounts between two groups, were presented as pair-wise comparisons using Metastats analysis. ** represents *P* < 0.01 compared with the CON group. A heat map of genera at the phylum (**h**) and family (**i**) levels were shown. Red represents upregulation and green represents downregulation. (**j**) The alpha diversity (observed species, Shannon, Simpson, ACE, Chao1, and Good’s coverage) of intestinal flora between CON group and AFLD group was shown. Cluster analysis result for both CON group and AFLD group according to NMDS (**k**) and Principal coordinate analysis (PCoA) (l). PCoA was performed using Bray-Curtis

**Fig. 2:**
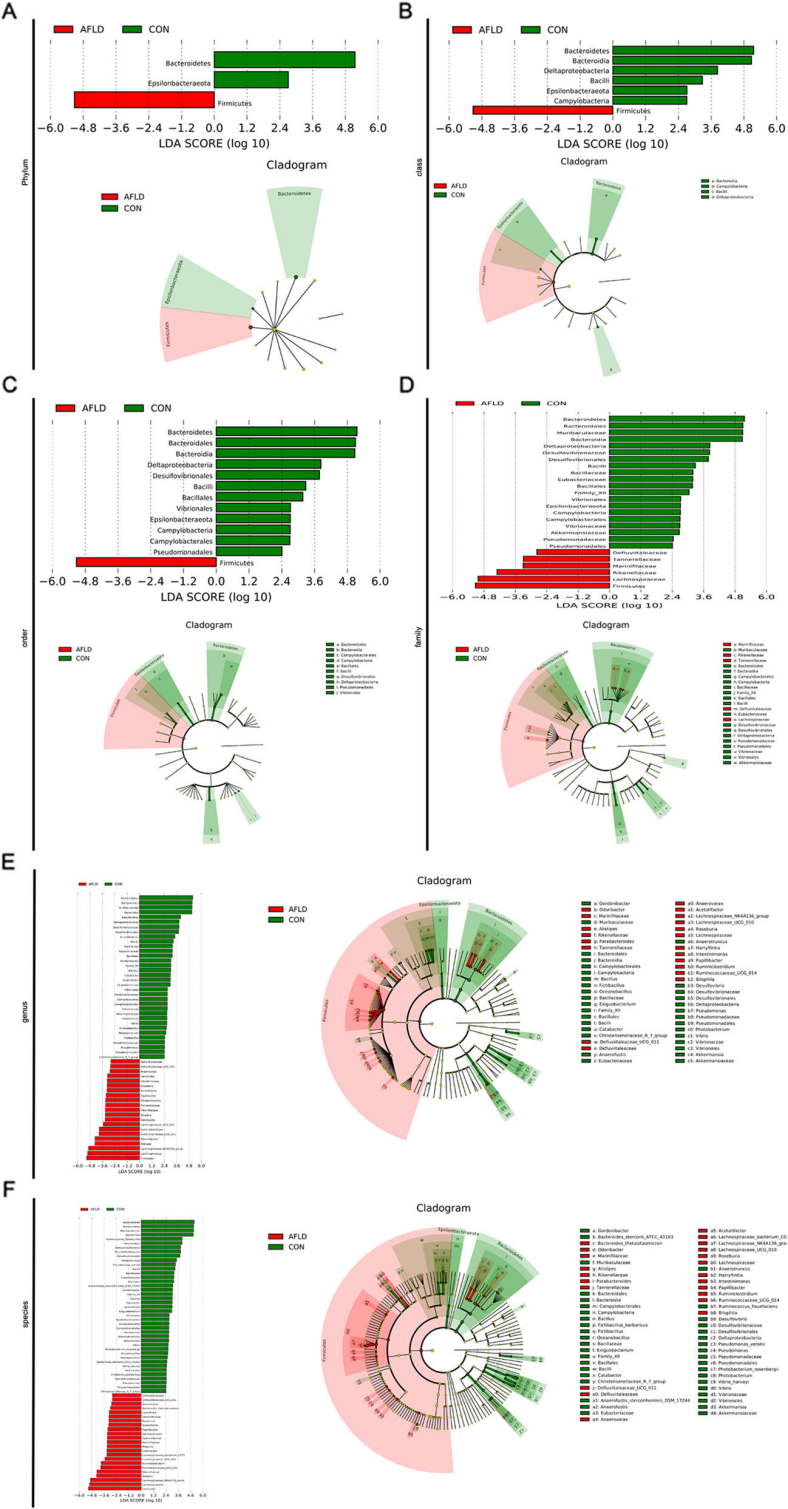
Results of LEFSE analysis. The histogram shows species whose LAD Score exceeded the default value of |2|. The length of the histogram represents the impact of the phylum (**a**), class (**b**), order (**c**), family (**d**), genus (**e**), or species (**f**). The different species have the same color as the group in the evolutionary branch diagram. Red represents AFLD group and green represents CON group

The microbiota can influence the development and progression of AFLD. High-throughput 16S rRNA gene sequencing produced a total of 25,253 good-quality sequences from 6 samples. These were grouped into 382 OTUs based on 97% similarity (Fig. 1a). A total of 285 OTUs were identified as core bacterial OTUs (Fig. 1b). We detected 7 bacterial phyla and the 60 genera among the mice. The structure and composition of the gut microbiota were significantly influenced by AFLD. The results of the top 10 most abundant OTUs at all taxonomic levels in samples, as inferred by GraPhlAn, showed that *Bacteroidetes*, *Firmicutes*, and *Proteobacteria* were the most abundant phyla (Fig. 1c), and *Muribaculaceae*, *Lachnospiraceae*, *Runminococcaceae*, and *Erysipelotrichaceae* were the most abundant family among the OTUs (Fig. 1f). Additionally, the relative bacterial abundance between the CON group and AFLD group at the phylum and family levels is shown in Fig. 1d and e, respectively. At the phylum level, the taxonomic profiles indicated that the proportions of *Bacteroidetes* and *Proteobacteria* decreased significantly, and the abundance of *Firmicutes* increased markedly in the AFLD group. The relative abundance ration of *Firmicutes* and *Bacteroidetes* increased significantly in the AFLD group. At the family level, the taxonomic profiles indicated that the proportions of *Muribaculaceae* decreased significantly, and the abundance of *Lachnospiraceae*, *Runminococcaceae*, and *Erysipelotrichaceae* increased markedly in the AFLD group (Fig. 1g). A heat map of genera indicated that the 1 genera (*Firmicutes*) increased significantly and 8 genera decreased significantly (Fig. 1h) at the phylum level, while 12 genera increased significantly, and the 8 genera decreased significantly (Fig. 1i) at the family level. The alpha diversity (observed species, Shannon, Simpson, ACE, Chao1, and Good’s coverage) of intestinal flora was calculated between the CON group and AFLD group in mice. The Shannon index was significantly lower in the AFLD group than in the CON group (PShannon = 0.04; Fig. 1j). In addition, there was no difference in the observed species index, Simpson index, ACE index, Chao1 index, and Good’s coverage index between the CON group and AFLD group. Multi-response permutation procedure (MRPP) testing between the CON group and AFLD group and the CON and AFLD groups yielded A > 0. The intergroup differences were larger than the intragroup differences, which further indicated that the grouping in this study was reasonable. Combined with the non-metric multi-dimensional scaling (NMDS) plot (Fig. 1k) and PCoA plots (Fig. 1l), it was shown that the CON and AFLD groups were significantly different. The results of the LDA effect size analysis showed phylum, class, order, family, genus, or species with significantly different abundance between the CON group and AFLD group. The results have two parts: the LDA value distribution histogram and the evolutionary branch diagram (phylogenetic distribution). *Bacteroidetes* is the most important phylum, class, order, family, genus, or species in the CON group, and *Firmicutes* is the most phylum, class, order, family, genus, or species in the AFLD group (Fig. 2).

### Prediction of gut microbiome function

**Fig. 3:**
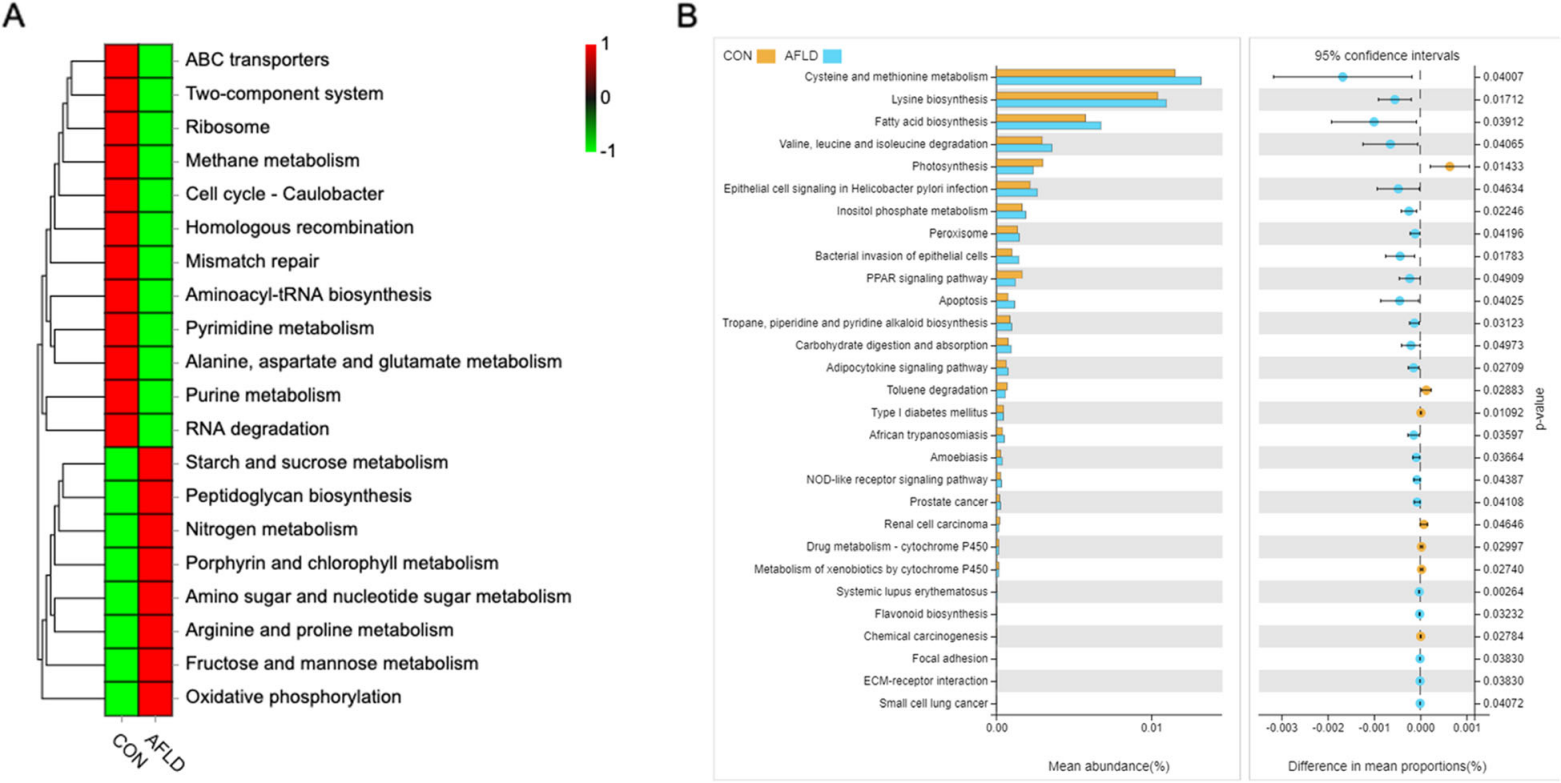
Analysis of gut microbiome function. **a** Relative abundance heat map of microbiota functions based on the KEGG database on the third level. **b** A total of 29 significantly changed the KEGG pathways in AFLD mice on the third level using Welch’s test. These data were obtained by Tax4Fun

The Tax4Fun analysis based on metagenomes was used to predict the functions of gut microbiota that were influenced by AFLD in mice. According to the results of the predicted function, the top 20 items of functional information of maximum abundance were selected for each sample of the KEGG classification level, and a relative functional abundance heat map was generated (Fig. 3a). At level 3, the results revealed that 29 KEGG pathways changed significantly between the AFLD group and CON group using Welch’s test (Fig. 3b). In particular, we found several interesting changes among the 29 altered KEGG pathways at level 3. The PPAR signaling pathway was increased in the CON group compared to that in the AFLD group, whereas, apoptosis was increased in the AFLD group compared to that in the CON group.

### Identification of DEGs and construction of PPI network in GSE8006 data set

**Fig. 4:**
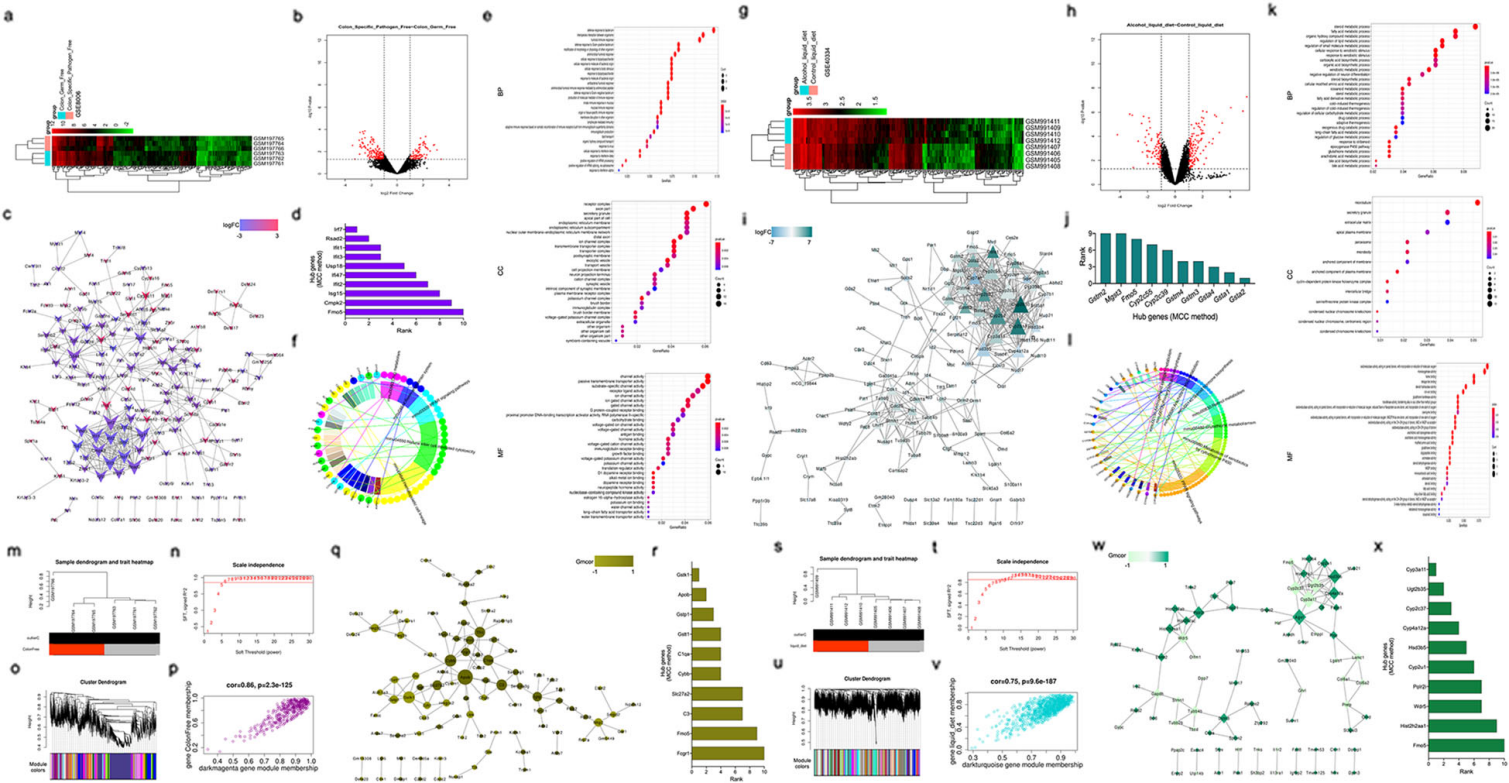
Identification of DEGs, construction of PPI network, and WGCNA analysis. For GSE8006 dataset, **a** The heat map for DEGs. **b** The volcano plot for DEGs. Black dots represent genes which are not differentially expressed, left dots represent the upregulated genes, and the right dots represent the downregulated genes. **c** A PPI network of DEGs was visualized by Cytoscape software. **d** The top 10 hub genes were identified by the cytohubba plug-in of Cytoscape software. GO terms, including BP, CC, and MF were shown in (**e**). **f** KEGG enrichment analysis was shown based on DAVID database. For GSE40334 dataset, **g** The heat map for DEGs. **h** The volcano plot for DEGs. Black dots represent genes which are not differentially expressed, left dots represent the upregulated genes, and the right dots represent the downregulated genes. **i** A PPI network of DEGs was visualized by Cytoscape software. **j** The top 10 hub genes were identified by the cytohubba plug-in of Cytoscape software. GO terms, including BP, CC, and MF were shown in (**k**). **l** KEGG enrichment analysis was shown based on DAVID database. **m** One outlier (GSE8006) was detected by sample clustering; **n** analysis of the scale-free topology model fit index for soft threshold powers (β) and the mean connectivity for soft threshold powers; **o** A cluster dendrogram was built based on the dissimilarity of the topological overlap, which presents 31 gene co-expression modules in colon specific pathogen free group; Each designated color represents a certain gene module; **p** The module relationships and significance; **q** A PPI network of the hug genes from in the darkmagenta module was visualized by Cytoscape software; **r** The top 10 hub genes of this PPI network were identified by the cytohubba plug-in of Cytoscape software. **s** One outlier (GSE40334) was detected by sample clustering; **t** analysis of the scale-free topology model fit index for soft threshold powers (β) and the mean connectivity for soft threshold powers; **u** A cluster dendrogram was built based on the dissimilarity of the topological overlap, which presents 111 gene co-expression modules in colon specific pathogen free group; Each designated color represents a certain gene module; **v** The module relationships and significance; **w** A PPI network of the hug genes from in the darkturquoise module was visualized by Cytoscape software; **x** The top 10 hub genes of this PPI network were identified by the cytohubba plug-in of Cytoscape software

A total of 292 DEGs (174 downregulated genes and 118 upregulated genes) in the GSE8006 dataset are shown in Fig. 4a and b, and were used to preform GO, and KEGG enrichment analysis and construct a PPI network using the DAVID and STRING database. Our results showed that the top 10 hub genes of a PPI network (Fig. 4c), including Fmo5, Cmpk2, Isg15, lfit2, lfit47, Usp18, lfit3, lfit1, Rsad2, and lrf7, were identified using cytohubba plug-in of Cytoscape software based on the “MCC” method (Fig. 4d). Next, 292 DEGs were mainly enriched in three GO terms, including BP, CC, and MF (Fig. 4e). KEGG enrichment analysis results showed that the PPAR signaling pathway was an important KEGG pathway (Fig. 4f).

### Identification of DEGs and construction of PPI network in GSE40334 data set

A total of 246 DEGs (98 downregulated genes and 148 upregulated genes) in GSE40334 dataset are shown in Fig. 4g and h, and were used to preform GO, and KEGG enrichment analysis and construct a PPI network using the DAVID and STRING databases. Our results showed that the top 10 hub genes of a PPI network (Fig. 4i), including Gstm2, Mgst3, Fmo5, Cyp2c55, Cyp2c39, Gstm4, Gstm3, Gsta4, Gsta1, and Gsta2, were identified using cytohubba plug-in of Cytoscape software based on the “MCC” method (Fig. 4j). Next, 246 DEGs were mainly enriched in three GO terms, including BP, CC, and MF (Fig. 4k). The KEGG enrichment analysis results showed that the PPAR signaling pathway was an important KEGG pathway (Fig. 4l).

### WGCNA analysis of GSE8006 and GSE40334 data sets

The WGCNA was performed to analyze the GSE8006 microarray data. First, the dataset was properly normalized and filtered to reduce outliers (Fig. 4m). The power value was screened out so that when the value was 6, the scale independence was 0.86 (Fig. 4n). Thus, this network conformed to the power-law distribution and was closer to the real biological network state. As shown in Fig. 4o, the cluster dendrogram contained 31 co-expression modules. The co-expressed genes were primarily clustered in the darkmagenta module. In order to explore the function of a module, we selected the darkmagenta module for further analysis. Our results indicated that the co-expression genes in the darkmagenta module were significantly associated with colon-specific pathogen-free (correlation coefficient = 0.86, *p* = 2.3e-125). Therefore, the darkmagenta module was a central module in all of the modules (Fig. 4p). A total of 181 hub genes in the darkmagenta module were identified. We also constructed a PPI network of the 181 hub genes using the STRING database, and Cytoscape software was used to visualize the PPI network (Fig. 4q). Next, the top 10 hub genes of a PPI network, including Fcgr1, Fmo5, C3, Slc27a2, Cybb, C1qa, Gstt1, Gstp1, Apob, and Gstk1, were identified using cytohubba plug-in of Cytoscape software based on the “MCC” method (Fig. 4r).

The WGCNA was performed to analyze the GSE40334 microarray data. First, the dataset was properly normalized and filtered to reduce outliers (Fig. 4s). The power value was screened out so that when the value was 11, the scale independence was 0.83 (Fig. 4t). Thus, this network conformed to the power-law distribution and was closer to the real biological network state. As shown in Fig. 4u, the cluster dendrogram contained 111 co-expression modules. The co-expressed genes were primarily clustered in the darkturquoise module. In order to explore the function of a module, we selected the darkturquoise module for further analysis. Our results indicated that the co-expression genes in the darkturquoise module were significantly associated with alcohol liquid diet (correlation coefficient = 0.75, *P* = 9.6e-187). Therefore, the darkturquoise module was a central module in all of the modules (Fig. 4v). A total of 172 hub genes in the darkturquoise module were identified. We also constructed a PPI network of the 172 hub genes using the STRING database, and Cytoscape software was used to perform the visualization of a PPI network (Fig. 4w). Next, the top 10 hub genes of a PPI network, including Fmo5, Hist2hg2aa1, Wdr5, Polr2i, Cyp2u1, Hsd3b5, Cyp4a12a, Cyp2c37, Ugt2b35, and Cyp3a11, were identified using cytohubba plug-in of Cytoscape software based on the “MCC” method (Fig. 4x).

### Fmo5 plays an important role in the AFLD-induced apoptosis and inflammatory response in the small intestine and liver tissues of mice and is closely related to PPARα expression and the NF-κB signaling pathway in vivo and in vitro

**Fig. 5:**
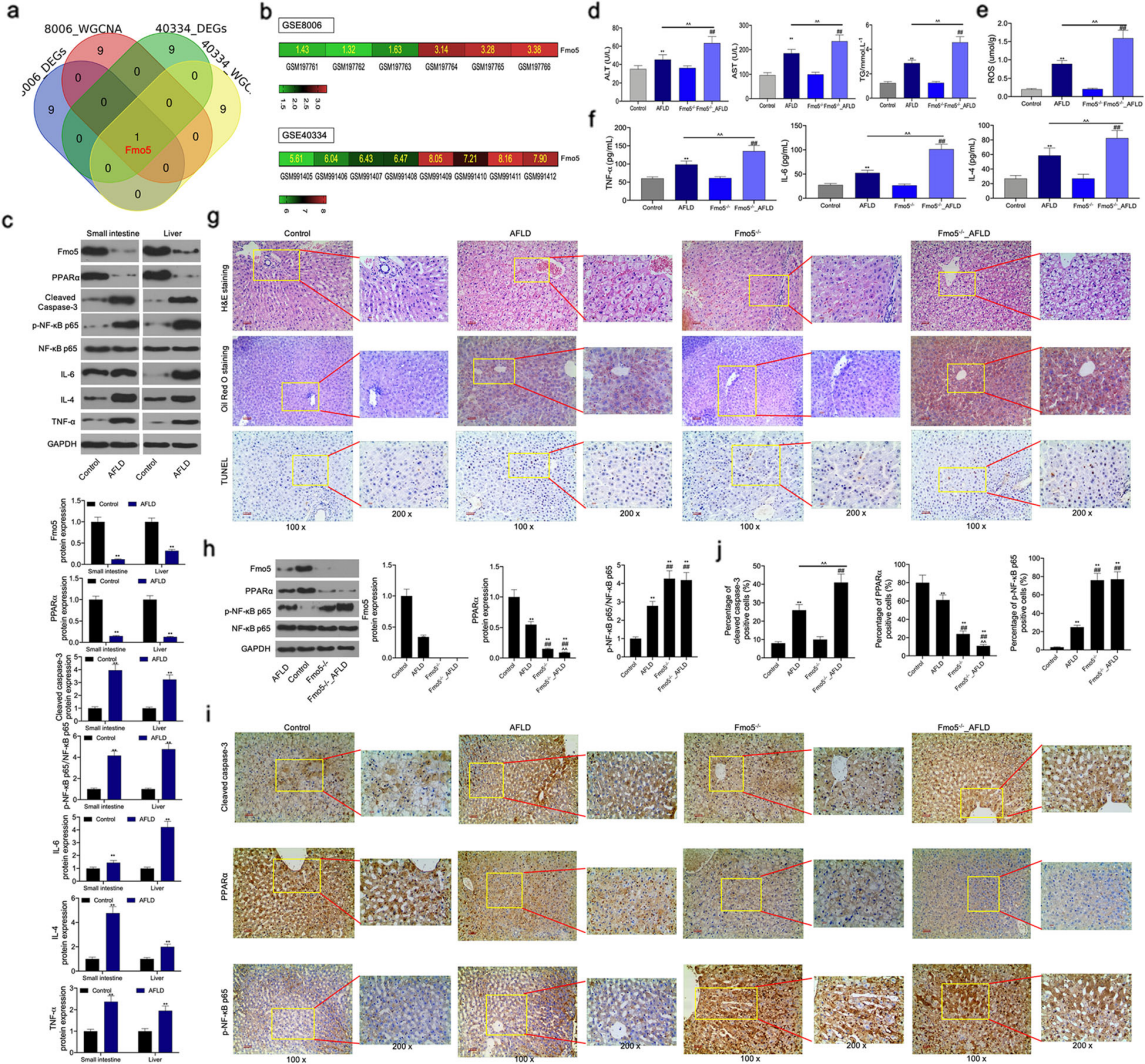
Fmo5 plays an important role in the AFLD-induced apoptosis and inflammatory response in the small intestine and liver tissues of mice and is closely related to PPARα expression and the NF-κB signaling pathway in vivo (**a**) Venn map of the one overlapping gene from the hub genes of the above 4 PPI network. **b** The heat map of Fmo5 expression in GSE8006 and GSE40334 datasets. **c** Western blot assay was used to detect the expression of Fmo5, PPARα, cleaved caspase-3, phospho-NF-κB p65, NF-κB p65, IL-6, IL-4, and TNF-α in the small intestine and liver tissues between the control group and the AFLD group. **d** ELISA assay was used to detect the levels of ALT, AST, and TG in blood sample between the control group and the AFLD group. **e** ELISA assay was used to detect ROS level in blood sample between the control group and the AFLD group. **f** ELISA assay was used to detect the levels of IL-6/− 4 and TNF-α in blood sample between the control group and the AFLD group. **g** H&E staining was used to observe the pathological change in liver tissues among the four group, Oil Red staining was used to observe the lipid deposition in liver tissues among the four group, and TUNEL was used to detect the apoptosis in liver tissues among the four group (magnification times: 100x and 200x; label with an arrow as to indicates a lipid deposit). **h** Western blot assay was used to detect the expression of Fmo5, PPARα, phospho-NF-κB p65 and NF-κB p65 in liver tissues among the four group. **i** IHC assay was detect the expression of PPARα, cleaved caspase-3, and phospho-NF-κB p65 in liver tissues among the four group (magnification times: 100x and 200x). GAPDH used as a load control. Data are presented as the mean ± standard deviation. ** *P* < 0.01 vs. Control group, ## *P* < 0.01 vs. AFLD group, and ^^ *P* < 0.01 vs. Fmo5−^/−^ group

Our results showed that Fmo5 was the only one overlapping gene from the hub genes of the 4 PPI network (Fig. 5a) and Fmo5 expression in GSE8006 and GSE40334 datasets was upregulated significantly (Fig. 5b). Furthermore, the protein levels of Fmo5 and PPARα in the small intestine and liver tissues of mice with AFLD decreased significantly, and the protein levels of cleaved caspase-3, IL-6, IL-4, and TNF-α, and the phosphorylation level of NF-κB p65 in the small intestine and liver tissues of mice with AFLD increased significantly (Fig. 5c). In order to explore the function of Fmo5 in AFLD, we selected Fmo5 knockout mice for further analysis. The results showed that Fmo5 knockout aggravated the AFLD-induced the abnormal liver function ROS production, and inflammatory response (Fig. 5d-f). H&E and Oil Red staining and TUNEL assay results showed that the degree of liver injury, lipid accumulation, and liver cell apoptosis in Fmo5 knockout mice with AFLD were much higher than those in AFLD mice (Fig. 5g). In addition, Fmo5 knockout inhibited PPARα expression and activated the NF-κB signaling pathway, as shown using western blot and IHC assays in mice with AFLD (Fig. 5h and i).

**Fig. 6:**
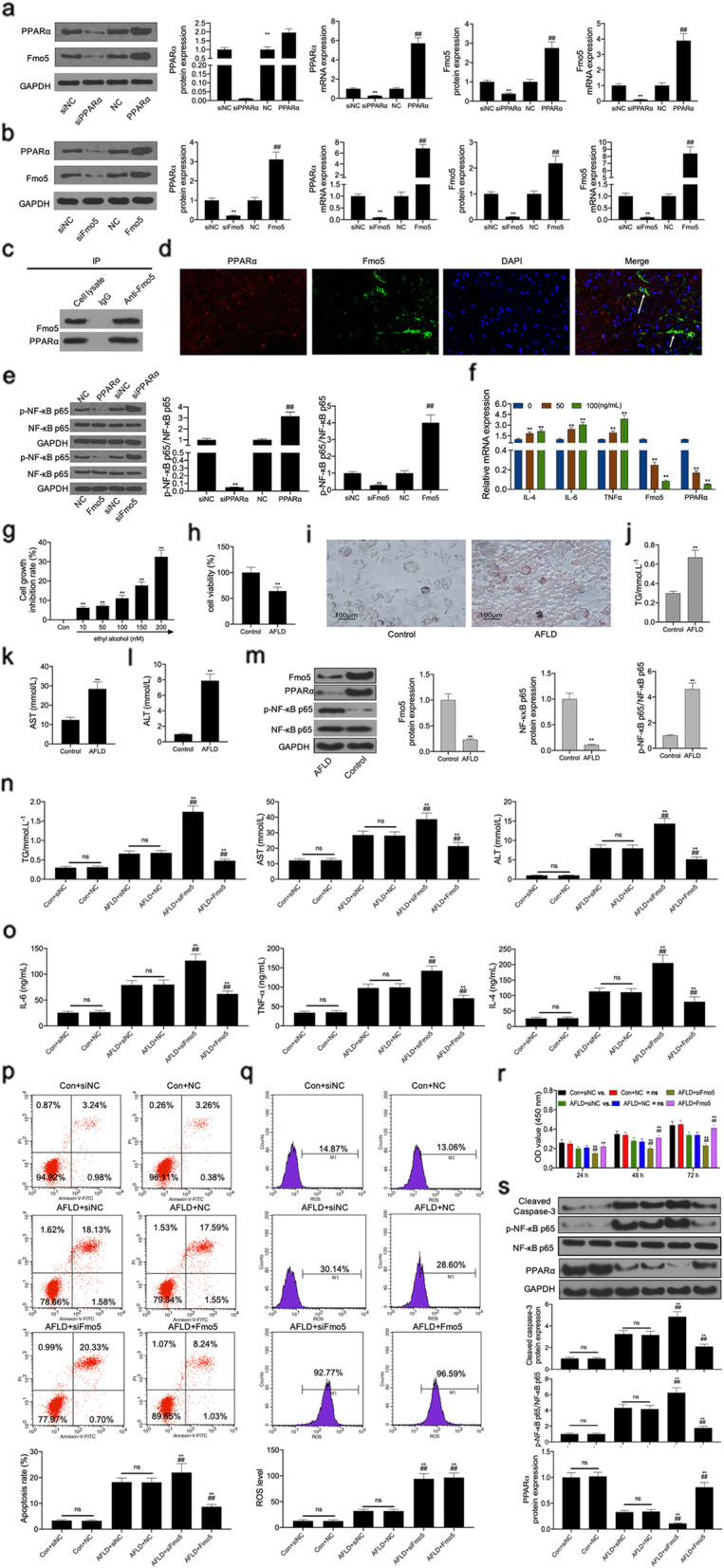
Fmo5 plays an important role in the AFLD-induced apoptosis and inflammatory response in the small intestine and liver tissues of mice and is closely related to PPARα expression and the NF-κB signaling pathway in vitro*.* **a** and **b** Western blot and RT-PCR assays were used to detect the protein and mRNA levels of Fmo5 and PPARα in siRNA targeting Fmo5/or PPARα transfected L02 cell or vector expressing Fmo5/or PPARα transfected L02 cells. **c** Co-immunoprecipitation experiments showed that Fmo5 directly interact with PPARα in L02 cell. **d** Double immunofluorescent staining of PPARα (red) and Fmo5 (green) showed colocalization of PPARα and Fmo5 in the L02 cells (indicated by a white arrow). **e** Western blot assay was used to detect the phosphorylation level of NF-κB p65 in siRNA targeting PPARα/or Fmo5 transfected L02 cell or vector expressing PPARα/or Fmo5 transfected L02 cells. **f** RT-PCR assay was used to detect the mRNA levels of IL-4/− 6, TNF-α, PPARα and Fmo5 in LPS treated L02 cells. **g** CCK-8 assay was used to detect the cell growth inhibition rate after L02 cells were treated with 0–200 nM of ethyl alcohol. **h** CCK-8 was used to detect the cell viability between the control and AFLD groups. AFLD cell model was shown in i. **j-l** ELISA assay was used to detect the levels of TG, AST, and ALT in L02 cells between the control and AFLD groups. (**m**) Western blot assay was used to detect the protein levels of Fmo5, PPARα, phospho-NF-κB p65 and NF-κB p65 in L02 cells between the control and AFLD groups. ELISA assay was used to detect TG, AST, ALT levels (**n**), IL-6, TNF-α, IL-4 levels (**o**) in L02 cells among the six groups. Flow cytometry assay was used to detect cell apoptosis (**p**) and ROS level (**q**) in L02 cells among the six groups. **r** CCK-8 assay was used to detect the viability of L02 cells among the six groups. **s** Western blot assay was used to detect the protein levels of cleaved caspase-3, phospho-NF-κB p65, NF-κB p65and PPARα in L02 cells among the six groups. GAPDH used as a load control. Data are presented as the mean ± standard deviation. Con+siNC vs. Con+NC = ns; AFLD+siNC vs. AFLD+NC = ns. ** *P* < 0.01 vs. siNC, control, Con+siNC and Con+NC groups, ## *P* < 0.01 vs. NC, AFLD+siNC and AFLD+NC groups

In vitro, we found that PPARα expression and Fmo5 in transfected L02 cells with PPARα overexpression increased significantly and that in siRNA targeting PPARα-transfected L02 cells decreased significantly, as shown using RT-PCR and western blot assays (Fig. 6a). Similarly, PPARα expression and Fmo5 in Fmo5 overexpression transfected L02 cells increased significantly and in siRNA targeting Fmo5 transfected L02 cells decreased significantly (Fig. 6b). Co-IP experiments indicated that Fmo5 directly interacts with PPARα in L02 cells (Fig. 6c). Double immunofluorescent staining results showed that both Fmo5 and PPARα were co-expressed in L02 cells (Fig. 6d). The results indicated that PPARα or Fmo5 overexpression inhibited the phosphorylation level of NF-κB p65 and PPARα or Fmo5 inhibition increased the phosphorylation level of NF-κB p65 (Fig. 6e). The mRNA levels of IL-4/− 6 and TNF-α increased significantly in a dose dependent manner and the mRNA levels of PPARα and Fmo5 decreased significantly in a dose dependent manner in LPS treated L02 cells (Fig. 6f). The CCK-8 results showed that ethyl alcohol inhibited cell growth in a concentration-dependent manner (Fig. 6g). Therefore, 200 nM was considered as the action concentration to induce AFLD in vitro. The cell ability in the AFLD group decreased significantly (Fig. 6h). Our results showed that the accumulation of lipid droplets was observed in the cytoplasm of L02 cells using Oil Red O staining (Fig. 6i). The levels of TG, AST, and ALT in the AFLD group increased significantly (Fig. 6j-l). This showed that the AFLD cell model was successfully constructed. In addition, the protein levels of Fmo5 and PPARα in the AFLD group decreased significantly and the phosphorylation level of NF-κB p65 in the AFLD group increased significantly (Fig. 6m). Next, the functions of AFLD, which inhibit cell viability, induce cell apoptosis, and increase the levels of TG, AST, ALT, IL-6, IL-4, TNFα, and ROS were upregulated by Fmo5 inhibition, whereas, these effects were reversed by Fmo5 overexpression (Fig. 6n-r). In addition, inhibition of Fmo5 upregulated cleaved caspase-3 expression, and the phosphorylation level of NF-κB p65 and inhibited PPARα expression in the AFLD cell model. Meanwhile, Fmo5 overexpression downregulated cleaved caspase-3 expression, and the phosphorylation level of NF-κB p65 and increased PPARα expression in the AFLD cell model (Fig. 6s).

### Fmo5 and PPARα co-expression alleviates AFLD-induced apoptosis, inflammatory response and the nuclear translocation of NF-κB p65 in vitro

**Fig. 7:**
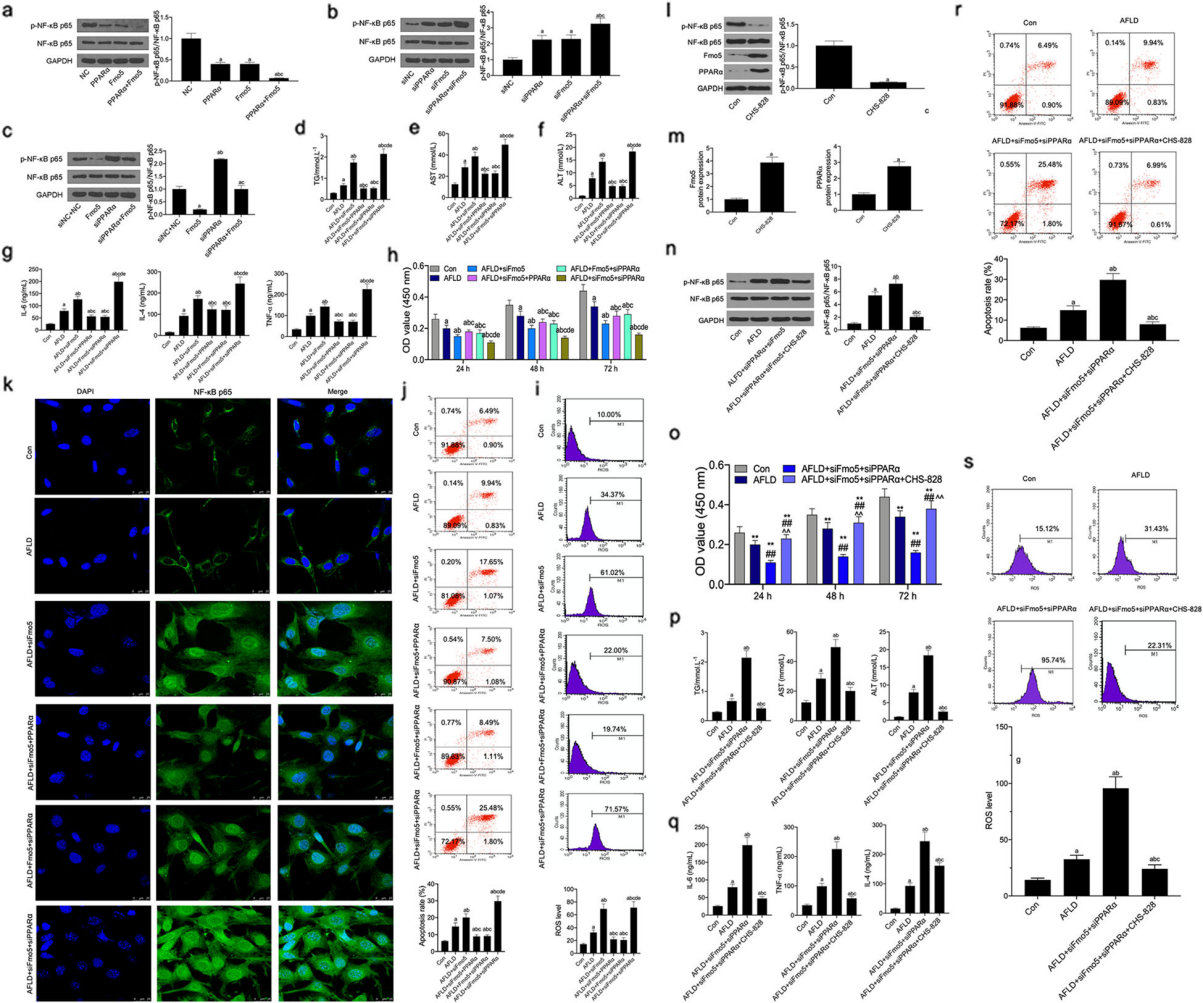
Fmo5 and PPARα co-expression alleviates AFLD-induced apoptosis, inflammatory response and the nuclear translocation of NF-κB p65 via inhibiting the activation of NF-κB signaling pathway in vitro*.* Western blot assay was used to detect the phosphorylation level of NF-κB p65 (**a**) in vector expressing PPARα and Fmo5 transfected L02 cells, **b** in siRNA targeting PPARα and Fmo5 transfected L02 cells, and (c) in siRNA targeting PPARα and vector expressing Fmo5. ELISA assay was used to detect TG level (**d**), AST level (**e**), ALT level (**f**), and IL-6/− 4 and TNF-α levels (**g**) in L02 cells among six groups. **h** CCK-8 assay was used to detect the cell viability in L02 cells among six groups. Flow cytometry assay was used to detect ROS level (**i**) and cell apoptosis (**j**) in L02 cells among the six groups. **k** Immunofluorescence staining was used to observe the nuclear translocation of NF-κB p65 in L02 cells among six groups. **l** and **m** Western blot assay was used to detect the protein levels of Fmo5, PPARα, phospho-NF-κB p65 and NF-κB p65 in CHS-828 treated L02 cells. After CHS-828 pre-treated with L02 cells with AFLD for 4 h, siRNA-Fmo5 and siRNA-PPARα were co-transfected into cells, the level of phospho-NF-κB p65 (**n**) was detected by Western blot assay, cell viability (**o**) was detected by CCK-8 assay, the levels of TG, AST, ALT (p), IL-6, IL-4, and TNF-α level (**q**) were detected by ELISA assay, cell apoptosis (**r**), and ROS level (**s**) were detected by Flow cytometry assay. Data are presented as the mean ± standard deviation. ^a^ *P* < 0.01 vs. NC/or siNC+NC/or Con group, ^b^ *P* < 0.01 vs. PPARα/or Fmo5/or siPPARα/or AFLD group, ^c^ *P* < 0.01 vs. Fmo5/or siFmo5/or siPPARα/or AFLD+siFmo5 group, ^d^ *P* < 0.01 vs. AFLD+siFmo5+ PPARα group, and ^e^ *P* < 0.01 vs. AFLD+Fmo5+ siPPARα group

The results showed that the phosphorylation level of NF-κB p65 in PPARα overexpression and Fmo5 overexpression co-transfected L02 cells was significantly lower than that in transfected L02 cells with PPARα overexpression and Fmo5 overexpression-transfected L02 cells (Fig. 7a). The phosphorylation level of NF-κB p65 in PPARα inhibition and Fmo5 inhibition co-transfected L02 cells was much higher than that in transfected L02 cells with PPARα overexpression and Fmo5 inhibition-transfected L02 cells (Fig. 7b). Next, western blot results showed that Fmo5 overexpression counteracted the function of PPARα silencing, increasing the phosphorylation level of NF-κB p65, and that PPARα silencing counteracted the function of Fmo5 overexpression, inhibiting the phosphorylation level of NF-κB p65 (Fig. 7c). The functions of Fmo5 silencing or PPARα silencing inhibiting ALFD-induced the inhibition of cell viability and cell apoptosis, and the elevation of TG, AST, ALT, IL-6, IL-4, TNFα, and ROS levels was abolished by PPARα overexpression or Fmo5 overexpression (Fig. 7d-j). Likewise, siRNA-Fmo5 and siRNA-PPARα co-transfection inhibited cell viability, induced cell apoptosis, and increased the levels of TG, AST, ALT, IL-6, TNFα, and ROS in the AFLD cell module. These functions in the AFLD+siFmo5 + siPPARα group were significantly higher than those in the AFLD+siFmo5 group. Immunofluorescence staining results proved that Fmo5 inhibition promoted AFLD-induced the nuclear translocation of NF-κB in L02 cells (Fig. 7k). However, PPARα overexpression reduced AFLD-induced the nuclear translocation of NF-κB p65 in siRNA-Fmo5-transfected L02 cells, while Fmo5 overexpression reduced AFLD-induced the nuclear translocation of NF-κB p65 in siRNA-PPARα-transfected L02 cells. In addition, the functions of co-transfection of siRNA-Fmo5 and siRNA-PPARα promoting the nuclear translocation of NF-κB p65 were significantly higher than that of siRNA-Fmo5 transfection in the AFLD cell model.

### Fmo5 and PPARα co-expression alleviates AFLD-induced apoptosis and inflammatory response via inhibiting the activation of NF-κB signaling pathway in vitro

CHS-828 (NF-κB-specific inhibitor) significantly inhibited the phosphorylation level of NF-κB p65 and significantly increased the protein levels of Fmo5 and PPARα (Fig. 7l-m). Alternatively, PPARα silencing and Fmo5 silencing aggravated AFLD-induced the high phosphorylation level of NF-κB p65, however, this function was abolished by CHS-828 (Fig. 7n). The CCK-8 assay showed that CHS-828 reversed the functions of the co-transfection of siRNA-Fmo5 and siRNA-PPARα promoting the inhibition of cell viability and the increase in TG, AST, ALT, IL-6, IL-4, and TNFα levels in the AFLD cell model (Fig. 7o-q). Flow cytometry analysis showed that the effects of co-transfection of siRNA-Fmo5 and siRNA-PPARα on promoting AFLD-induced cell apoptosis and ROS levels were abolished by CHS-828 (Fig. 7r and s).

## Discussion

Inflammation increases intestinal osmotic pressure, causing endotoxin to cross the intestinal barrier and enter the body, forming a vicious circle. The results of 16S rDNA sequencing indicated that the ratio of *Firmicutes/Bacteroidetes* in the gut of mice with AFLD was higher than that in the gut of normal mice and the abundance of bacteria in the gut of mice with AFLD was lower than that in the gut of normal mice. These data showed that AFLD induced gut microbiota disturbance. Intestinal flora can directly act on intestinal epithelial cells, affect their gene expression, and then affect intestinal function and the activation of related signaling pathways. Disturbance of intestinal flora can further damage the intestinal barrier and spread alcohol-related damage to liver tissue [17]. There is a close relationship between intestine and liver in anatomy and function, that is, the intestinal-liver axis [18]. With the decrease in liver function, the intestinal balance is destroyed. With the decrease of liver function, the intestinal balance is destroyed [19]. The accumulation of harmful substances such as alcohol leads to liver injury and promotes the secretion of bile acid into the intestinal tract by the liver, thereby regulating the activity of various metabolic reactions in the intestinal tract [2, 4, 5, 13]. The change in the type and number of intestinal flora is an important factor leading to abnormal liver function [20]. Liver disease can also lead to overgrowth and imbalance of intestinal flora [5, 17]. Long-term drinking will cause intestinal flora to release endotoxins and destroy the intestinal barrier, causing excessive production of inflammatory factors (such as IL-6, IL-4, and TNF-α), and promoting the occurrence and development of AFLD [21]. Therefore, the expression of bacterial genes related to inflammation and alcohol metabolism will change significantly in the intestines and liver of patients with AFLD. Our results showed that gut microbiome function was significantly related to the PPAR signaling pathway and apoptosis. The results of bioinformatics analysis showed that Fmo5 was the only hub gene, significantly associated with intestinal microorganisms and alcoholic diet, which had a protective effect on the liver. Interestingly, there is a co-expression relationship between Fmo5 and PPARα both in vivo and in vitro. However, Fmo5 was not expressed in Fmo5 ^−/−^ mice and PPARα was still expressed in Fmo5 ^−/−^ mice. This could be due to the Fmo5 knockout sequence that will nearly but not completely matched with the PPAR sequence. Therefore, PPAR cannot be completely silenced in Fmo5 ^−/−^ mice. PPARα is a key gene in the PPAR signaling pathway, which plays a role in cellular lipids by regulating the expression of genes involved in lipid metabolism in the liver [22, 23]. PPARα gene knockout promotes inflammation and aggravates lipid deposition in the liver. It can be seen that Fmo5 and PPARα are beneficial to the metabolic function of the liver. It has been reported that NF-κB and some proinflammatory markers (IL-6/− 4 and TNF-α) were inhibited by PPARα, indicating that activated PPARα reduces the inflammatory response by inhibiting the NF-κB signaling pathway [24]. It is known that PPARα interferes with NF-κB involving direct protein-protein interaction with subunit p65, thus diminishing NF-κB DNA binding. NF-κB is an upstream signal molecule of many inflammatory mediators and is involved in apoptosis, which can promote the production of many inflammatory mediators and directly participate in acute and chronic inflammation of the liver [25, 26]. Studies have shown that in the process of establishing a model of alcoholic fatty liver, NF-κB is consistent with the degree of steatosis in liver tissue at the gene transcriptional level. The level of NF-κB in the serum of patients with alcoholic fatty liver is positively correlated with the indices of serum liver function. NF-κB is involved in the occurrence and development of alcoholic fatty liver [27–29]. Inflammation induces the production of free radicals or decreases the ability of the body to scavenge free radicals, thus prompting the body to release a large amount of ROS [30]. ROS can further activate inflammatory responses by activating the NF-κB signaling pathway [31]. The results of animal experiments show that Fmo5 and PPARα expression in the small intestine and liver tissues of mice with AFLD decreased significantly, and the expression of apoptosis-related proteins (cleaved caspase-3), phospho-NF-κB p65, and inflammation-associated cytokines (IL-6/− 4 and TNF-α) in in the small intestine and liver tissues of mice with AFLD increased significantly. In summary, AFLD induces intestinal flora imbalance, reduces the expression of intestinal-liver protection related factor (Fmo5 and PPARα), activates the NF-κB signaling pathway, and promotes apoptosis and inflammation. However, the anti-inflammatory and anti-apoptotic effects of Fmo5 in the liver need to be further studied.

Fmo5 gene knockout can significantly aggravate AFLD-mediated liver injury, lipid accumulation and apoptosis, promote the release of inflammatory cytokines, and activate the NF-κB signaling pathway. In addition, PPARα expression in the liver tissue of Fmo5^−/−^ mice and Fmo5^−/−^ mice with AFLD decreased significantly. The above results further suggest that AFLD activates the NF-κB signal pathway by inhibiting the expression of Fmo5 and PPARα, thus promoting inflammation and apoptosis and damaging liver tissue. The results of cell experiments showed that Fmo5 and PPARα were co-expressed in liver cells and Fmo5 and PPARα overexpression inhibited the phosphorylation level of NF-κB p65. It has been reported that LPS activates the NF-κB signal pathway [32]. We found that LPS increased the mRNA levels of IL-4/− 6 and TNF-α and decreased the mRNA levels of PPARα and Fmo5. These results showed that Fmo5/PPARα/ NF-κB axis exist in liver cells. Furthermore, AFLD promoted the nuclear translocation of NF-κB p65 by upregulating the phosphorylation level of NF-κB p65, thus activating the NF-κB signaling pathway. However, silencing of Fmo5 and PPARα can aggravate the role of Fmo5 silencing in promoting AFLD-induced apoptosis, inflammatory response, upregulation of ROS level and activation of the NF-κB signaling pathway. Rescue experiments showed that the co-expression of Fmo5 and PPARα in liver tissue has a protective effect on AFLD-mediated liver injury, which is closely related to the inhibition of the activation of the NF-κB signaling pathway.

## Conclusion

Fmo5 and PPARα are significantly downregulated in the liver tissue in AFLD and are closely related to intestinal flora and alcoholic diet. AFLD induces intestinal flora imbalance and reduces the expression of intestinal-liver protective factor α (Fmo5 and PPARα), thus activating the NF-κB signaling pathway, thereby promoting apoptosis and inflammation. Co-expression of Fmo5 and PPARα reduces AFLD-mediated apoptosis, inflammation and liver injury by inhibiting the activation of the NF-κB signaling pathway.

## Supplementary Information

**Additional file 1.**

**Additional file 2.**

## Data Availability

The datasets generated/analyzed during the current study are available.

## Abbreviations

DAVID: The Database for Annotation, Visualization and Integrated Discovery
PPI network: Protein-protein interaction network
STRING: The Search Tool for the Retrieval Interacting Genes
KEGG: Kyoto Encyclopedia of Genes and Genomes
MCC: Maximal clique centrality
GEO database: Gene Expression Omnibus database
DEGs: The differentially expression genes
GO: Gene Ontology
AFLD: Alcoholic fatty liver disease
WGCNA: Weighted gene co-expression network analysis
FMOs: Flavin containing monooxygenases
Fmo5: Flavin containing monooxygenase 5
