# Supplementary material for: Alcoholic fatty liver disease inhibited the co-expression of Fmo5 and PPARα to activate the NF-κB signaling pathway, thereby reducing liver injury via inducing gut microbiota disturbance
Source: J Exp Clin Cancer Res. 2021 Jan 7;40:18. doi: 10.1186/s13046-020-01782-w (PMC7788704; doi:10.1186/s13046-020-01782-w)
Supplement: Supplementary file 1 — Additional file 1. [file 13046_2020_1782_MOESM1_ESM.docx]

The supplementary information 1: DEGs in GSE8006 dataset

| ID | Genesymbol | logFC | P.Value |
| --- | --- | --- | --- |
| 1 | Igkv2-112 | -3.4229436 | 0.00275167 |
| 2 | Kap | -3.2474945 | 0.04812352 |
| 3 | Tfrc | -2.9087502 | 0.0009346 |
| 4 | Ifi47 | -2.8785302 | 0.01704466 |
| 5 | Arhgap29 | -2.8569137 | 0.00354932 |
| 6 | Dynlt1b | -2.8473933 | 0.00018476 |
| 7 | Slfn2 | -2.8355412 | 0.00239017 |
| 8 | Slfn3 | -2.781459 | 0.00338147 |
| 9 | Igkv1-133 | -2.7315457 | 0.00466745 |
| 10 | Igk-V1 | -2.6997764 | 0.00074241 |
| 11 | Igkv9-120 | -2.6997764 | 0.00074241 |
| 12 | Igkv4-68 | -2.6953818 | 0.00148638 |
| 13 | Napsa | -2.6519627 | 0.00147524 |
| 14 | Myh4 | -2.6372971 | 0.00016001 |
| 15 | Slfn4 | -2.6125446 | 0.00186989 |
| 16 | Ifi27l2a | -2.5953653 | 0.00374031 |
| 17 | Ace2 | -2.5798255 | 0.00023857 |
| 18 | Usp18 | -2.4985423 | 0.0030746 |
| 19 | Ifit2 | -2.4794599 | 0.00013766 |
| 20 | Gm9706 | -2.4729591 | 0.00056172 |
| 21 | C78878 | -2.4675375 | 0.00386323 |
| 22 | Cck | -2.4581735 | 0.01339511 |
| 23 | Ifit3 | -2.4301029 | 0.00475816 |
| 24 | Cwf19l1 | -2.4163054 | 0.00471644 |
| 25 | Igkv10-96 | -2.282903 | 0.00646766 |
| 26 | Hoxa4 | -2.2573203 | 0.02602656 |
| 27 | Slc7a9 | -2.2464119 | 0.00247661 |
| 28 | Ifit1 | -2.2073732 | 0.02053139 |
| 29 | Cr2 | -2.2070454 | 0.00739984 |
| 30 | Gp49a | -2.1236846 | 0.04884907 |
| 31 | Lilrb4 | -2.1236846 | 0.04884907 |
| 32 | Mme | -2.1183488 | 0.01189087 |
| 33 | Prm1 | -2.1130134 | 0.03707491 |
| 34 | Gbp3 | -2.094979 | 0.01614966 |
| 35 | Fabp1 | -2.0926633 | 0.02909029 |
| 36 | Igk-V28 | -2.0864313 | 0.00931487 |
| 37 | Fcer2a | -2.0619459 | 0.0050415 |
| 38 | Vipr2 | -2.0561985 | 0.00152214 |
| 39 | Tgtp1 | -2.0471319 | 0.00118506 |
| 40 | Tgtp2 | -2.0471319 | 0.00118506 |
| 41 | Isg15 | -2.0450501 | 0.00499596 |
| 42 | Mep1b | -1.9805194 | 0.02349871 |
| 43 | Tnfsf8 | -1.9761819 | 0.00050173 |
| 44 | Chrna1 | -1.9691305 | 0.00883395 |
| 45 | Ros1 | -1.9658084 | 0.01207336 |
| 46 | Zfy1 | -1.9568695 | 0.03181018 |
| 47 | Zfy2 | -1.9568695 | 0.03181018 |
| 48 | Apob | -1.9554559 | 0.00081504 |
| 49 | Cmpk2 | -1.9533435 | 0.01594096 |
| 50 | Trav9d-3 | -1.9509644 | 0.0309115 |
| 51 | Igkv14-111 | -1.8979706 | 0.00219146 |
| 52 | Slc34a2 | -1.8705327 | 0.03627892 |
| 53 | Igtp | -1.8554689 | 0.00108879 |
| 54 | Abcc6 | -1.8533593 | 0.00879039 |
| 55 | Tmem71 | -1.8505677 | 0.00725826 |
| 56 | Gabrr1 | -1.844165 | 0.00072869 |
| 57 | Vapa | -1.7993883 | 0.02816706 |
| 58 | Prl3c1 | -1.7928372 | 0.00087041 |
| 59 | Rsad2 | -1.7911416 | 0.01131498 |
| 60 | Klrk1 | -1.7674845 | 0.00143981 |
| 61 | LOC101056278 | -1.7666983 | 0.02736527 |
| 62 | C78344 | -1.7558268 | 0.00864376 |
| 63 | Amy1 | -1.7525016 | 0.01261774 |
| 64 | Irgm2 | -1.7349962 | 0.02912089 |
| 65 | Xdh | -1.7181138 | 0.04508065 |
| 66 | Rrh | -1.7118569 | 0.03056898 |
| 67 | Irgm1 | -1.699051 | 0.01159654 |
| 68 | Ptpn11 | -1.6599167 | 0.00581831 |
| 69 | Enpp2 | -1.6592129 | 0.02961996 |
| 70 | Plin4 | -1.6519856 | 0.03361949 |
| 71 | Npas1 | -1.6296524 | 0.01526968 |
| 72 | Nxn | -1.6121416 | 0.03933997 |
| 73 | Myoz1 | -1.6007362 | 0.02651765 |
| 74 | Ptch2 | -1.6004311 | 0.01807829 |
| 75 | Tspan5 | -1.597508 | 0.00786597 |
| 76 | Mcpt2 | -1.5701859 | 0.02064702 |
| 77 | Igkv15-103 | -1.5672839 | 0.01274495 |
| 78 | Homer1 | -1.5659919 | 0.04281917 |
| 79 | Neurod6 | -1.5611074 | 0.02632107 |
| 80 | Igkv4-70 | -1.5570373 | 0.02749141 |
| 81 | LOC434035 | -1.5453121 | 0.01176605 |
| 82 | Nufip1 | -1.5299212 | 0.04338079 |
| 83 | Kcnu1 | -1.5222526 | 0.01052875 |
| 84 | Tgfbi | -1.512478 | 0.01612833 |
| 85 | Cd5 | -1.5108935 | 0.0098246 |
| 86 | Igkv16-104 | -1.5064568 | 0.04557636 |
| 87 | Bdnf | -1.5030454 | 0.00398613 |
| 88 | Irf7 | -1.5000532 | 0.03738293 |
| 89 | Slc27a2 | -1.4897154 | 0.00298694 |
| 90 | Cd5l | -1.4708995 | 0.04348011 |
| 91 | Mid1 | -1.4690951 | 0.03145593 |
| 92 | Ly6e | -1.4672009 | 0.00917894 |
| 93 | Cd44 | -1.4661831 | 0.03973286 |
| 94 | Sirt1 | -1.4608098 | 0.0317767 |
| 95 | Osbpl9 | -1.4499803 | 0.02642683 |
| 96 | Ptbp2 | -1.4463704 | 0.04866418 |
| 97 | Ckmt2 | -1.4318186 | 0.0264466 |
| 98 | Pomc | -1.43125 | 0.03586721 |
| 99 | Cyp2b13 | -1.4180586 | 0.02009786 |
| 100 | Irf4 | -1.4150644 | 0.03424025 |
| 101 | Akap12 | -1.4121192 | 0.02963666 |
| 102 | Rps14 | -1.4068235 | 0.02678397 |
| 103 | 2610020H08Rik | -1.4063624 | 0.01654922 |
| 104 | Ndufa12 | -1.4025589 | 0.00645154 |
| 105 | Itgae | -1.390095 | 0.0114503 |
| 106 | Lonp1 | -1.3853731 | 0.03631998 |
| 107 | Igkv4-91 | -1.3695352 | 0.04793313 |
| 108 | Syt17 | -1.3621284 | 0.04343081 |
| 109 | Nrl | -1.3603067 | 0.04777759 |
| 110 | Gapdhs | -1.359776 | 0.04776881 |
| 111 | Lgr5 | -1.3587766 | 0.02914869 |
| 112 | Snx17 | -1.3577076 | 0.01386117 |
| 113 | C78513 | -1.3508052 | 0.03985844 |
| 114 | LOC382693 | -1.348352 | 0.0287735 |
| 115 | Keap1 | -1.3481934 | 0.00540897 |
| 116 | Krt16 | -1.3427301 | 0.03349621 |
| 117 | Prf1 | -1.3372478 | 0.01099294 |
| 118 | Igfbpl1 | -1.3307754 | 0.02260381 |
| 119 | Plp1 | -1.3268455 | 0.01478111 |
| 120 | Iglc1 | -1.321045 | 0.03567382 |
| 121 | Iglv1 | -1.321045 | 0.03567382 |
| 122 | Ppp1r1a | -1.3179751 | 0.04063692 |
| 123 | Eny2 | -1.302035 | 0.04271512 |
| 124 | Rspo1 | -1.2995009 | 0.04933588 |
| 125 | Rassf2 | -1.2941507 | 0.03938721 |
| 126 | Il2rg | -1.2912314 | 0.01376544 |
| 127 | Serpinb2 | -1.2759319 | 0.01210316 |
| 128 | Park2 | -1.271321 | 0.02011708 |
| 129 | Tcf7 | -1.243408 | 0.01628711 |
| 130 | Ak1 | -1.232843 | 0.04916669 |
| 131 | Igkv7-33 | -1.2251924 | 0.01004799 |
| 132 | Kcnd1 | -1.2248776 | 0.03306858 |
| 133 | Ighv8-12 | -1.2233378 | 0.04446538 |
| 134 | Ighv8-13 | -1.2233378 | 0.04446538 |
| 135 | Ighv8-9 | -1.2233378 | 0.04446538 |
| 136 | Cox6c | -1.2201148 | 0.03996544 |
| 137 | Cd52 | -1.2158476 | 0.04602979 |
| 138 | Folh1 | -1.2146187 | 0.02382206 |
| 139 | Elavl4 | -1.2145246 | 0.04120608 |
| 140 | 2310014L17Rik | -1.2117851 | 0.04710046 |
| 141 | Aif1 | -1.2086738 | 0.04915554 |
| 142 | Mt2 | -1.2054571 | 0.0108686 |
| 143 | Znfx1 | -1.1923046 | 0.02290443 |
| 144 | Foxf2 | -1.1876689 | 0.03487285 |
| 145 | Lmbr1l | -1.1683455 | 0.04257471 |
| 146 | Pdyn | -1.1575686 | 0.01216365 |
| 147 | Hcrt | -1.1519052 | 0.04716816 |
| 148 | Fbxl12 | -1.1488663 | 0.04330047 |
| 149 | Trib3 | -1.1488377 | 0.03998175 |
| 150 | Tef | -1.1431014 | 0.02264644 |
| 151 | Ptprz1 | -1.1368884 | 0.04739462 |
| 152 | Kcnj11 | -1.1317856 | 0.01940175 |
| 153 | Anpep | -1.1304596 | 0.0218779 |
| 154 | Rpl39 | -1.1296554 | 0.01887267 |
| 155 | Foxb1 | -1.1129448 | 0.03770467 |
| 156 | Klra1 | -1.1077544 | 0.0495548 |
| 157 | Klra15 | -1.1077544 | 0.0495548 |
| 158 | Klra18 | -1.1077544 | 0.0495548 |
| 159 | Klra4 | -1.1077544 | 0.0495548 |
| 160 | Dlg4 | -1.0991216 | 0.03699702 |
| 161 | Pfn2 | -1.0843639 | 0.0224539 |
| 162 | Calb1 | -1.0826425 | 0.03218962 |
| 163 | Cd79b | -1.0789748 | 0.01417116 |
| 164 | Tcn2 | -1.0694716 | 0.03527245 |
| 165 | Afp | -1.0666292 | 0.01794091 |
| 166 | Aqp2 | -1.0654908 | 0.0297677 |
| 167 | Ms4a6c | -1.0615734 | 0.04653065 |
| 168 | Lhcgr | -1.0486885 | 0.01440006 |
| 169 | Kcna4 | -1.0422854 | 0.03258072 |
| 170 | Krtap3-2 | -1.0383298 | 0.04995551 |
| 171 | Krtap3-3 | -1.0383298 | 0.04995551 |
| 172 | Col7a1 | -1.0271727 | 0.03151587 |
| 173 | Rpl32 | -1.0238455 | 0.03088488 |
| 174 | Emc3 | -1.0202782 | 0.0268788 |
| 175 | E130012A19Rik | 1.00463759 | 0.03707121 |
| 176 | Pvt1 | 1.01129882 | 0.04382907 |
| 177 | Elp5 | 1.01495675 | 0.02622044 |
| 178 | Arnt2 | 1.02719281 | 0.02000292 |
| 179 | Rarg | 1.03285386 | 0.04612535 |
| 180 | Kifc1 | 1.06566266 | 0.02591127 |
| 181 | Gm10256 | 1.08010478 | 0.02562192 |
| 182 | Gm10352 | 1.08010478 | 0.02562192 |
| 183 | Gm21677 | 1.08010478 | 0.02562192 |
| 184 | Gm21693 | 1.08010478 | 0.02562192 |
| 185 | Gm21704 | 1.08010478 | 0.02562192 |
| 186 | Gm21708 | 1.08010478 | 0.02562192 |
| 187 | Gm3376 | 1.08010478 | 0.02562192 |
| 188 | Gm4064 | 1.08010478 | 0.02562192 |
| 189 | Rbmy | 1.08010478 | 0.02562192 |
| 190 | Mip | 1.08164168 | 0.02836672 |
| 191 | Cd8a | 1.15085526 | 0.02638374 |
| 192 | Sorl1 | 1.18295621 | 0.04241118 |
| 193 | Msx2 | 1.19064695 | 0.04442864 |
| 194 | Syngr1 | 1.21256121 | 0.02454266 |
| 195 | Areg | 1.22448806 | 0.04278749 |
| 196 | S100a5 | 1.2269412 | 0.041239 |
| 197 | Cacna1e | 1.24034166 | 0.01491922 |
| 198 | Nkain1 | 1.24137481 | 0.01032331 |
| 199 | Ercc1 | 1.24882225 | 0.02800903 |
| 200 | Defa-ps1 | 1.27181545 | 0.03857059 |
| 201 | Fancc | 1.28307428 | 0.02419903 |
| 202 | Stk36 | 1.31235511 | 0.0361684 |
| 203 | Tectb | 1.34012446 | 0.02145717 |
| 204 | Gabrr2 | 1.34524765 | 0.04849552 |
| 205 | Plk-ps1 | 1.3959619 | 0.03011745 |
| 206 | Drd3 | 1.39614712 | 0.00644909 |
| 207 | Gpd1 | 1.41652575 | 0.04449428 |
| 208 | Tk1 | 1.43065553 | 0.04212916 |
| 209 | D8Ertd28e | 1.44423598 | 0.04484818 |
| 210 | Ggta1 | 1.44754086 | 0.02036412 |
| 211 | Akr1b8 | 1.44777377 | 0.00922993 |
| 212 | C76336 | 1.44985335 | 0.02736016 |
| 213 | Meis3 | 1.45324395 | 0.04016655 |
| 214 | D7Bwg0826e | 1.45559411 | 0.04823564 |
| 215 | Acvr1 | 1.47007092 | 0.03023893 |
| 216 | Obp1b | 1.47584184 | 0.02979778 |
| 217 | Mug1 | 1.52715514 | 0.02354067 |
| 218 | Kcna7 | 1.53731154 | 0.00733175 |
| 219 | Cpeb1 | 1.53823662 | 0.02227455 |
| 220 | C77681 | 1.53910691 | 0.02664939 |
| 221 | Traf1 | 1.54311887 | 0.00907252 |
| 222 | Papolb | 1.56192887 | 0.04255112 |
| 223 | Myl4 | 1.58237603 | 0.04228746 |
| 224 | AA517631 | 1.58687335 | 0.01373298 |
| 225 | Igh | 1.61024223 | 0.02579895 |
| 226 | Ighg1 | 1.61024223 | 0.02579895 |
| 227 | Hspa1l | 1.61919667 | 0.01287505 |
| 228 | Scn10a | 1.62313283 | 0.0392601 |
| 229 | Tubb2a-ps2 | 1.62789452 | 0.0373875 |
| 230 | Kazald1 | 1.628874 | 0.00989128 |
| 231 | Vnn1 | 1.63454045 | 0.04367191 |
| 232 | Prl2b1 | 1.66791738 | 0.0110669 |
| 233 | Smok3a | 1.6696949 | 0.04275353 |
| 234 | Smok3b | 1.6696949 | 0.04275353 |
| 235 | Smok3c | 1.6696949 | 0.04275353 |
| 236 | Ybx2 | 1.67399593 | 0.002371 |
| 237 | Srms | 1.67625619 | 0.02331684 |
| 238 | Etv2 | 1.68165753 | 0.0048355 |
| 239 | Pdc | 1.71145781 | 0.00978254 |
| 240 | Ccne2 | 1.731917 | 0.01774801 |
| 241 | Elavl2 | 1.73411549 | 0.02154001 |
| 242 | Zp3r | 1.73677267 | 0.04912136 |
| 243 | Crlf1 | 1.73683381 | 0.00661666 |
| 244 | Stx1a | 1.74453989 | 0.00250307 |
| 245 | Gstm6 | 1.75048712 | 0.03972188 |
| 246 | Ang3 | 1.75607774 | 0.00406217 |
| 247 | Ang5 | 1.75607774 | 0.00406217 |
| 248 | Defa2 | 1.77880463 | 0.00216618 |
| 249 | Defa20 | 1.77880463 | 0.00216618 |
| 250 | Gm15308 | 1.77880463 | 0.00216618 |
| 251 | Gm21002 | 1.77880463 | 0.00216618 |
| 252 | Fmo5 | 1.80228139 | 0.0006241 |
| 253 | Clec4d | 1.81412111 | 0.0314447 |
| 254 | LOC102047627 | 1.82666277 | 0.03269657 |
| 255 | Sprr1a | 1.83699505 | 0.0208981 |
| 256 | Krt81 | 1.86755483 | 0.01955577 |
| 257 | Crisp3 | 1.86906807 | 0.02956062 |
| 258 | Ptpn22 | 1.91829583 | 0.04935776 |
| 259 | Mycn | 1.94799591 | 0.02653007 |
| 260 | Spp1 | 1.95272806 | 0.02450285 |
| 261 | AI118078 | 1.97240053 | 0.00165735 |
| 262 | Defa-rs7 | 1.99966735 | 0.00253813 |
| 263 | Defa1 | 1.99966735 | 0.00253813 |
| 264 | Defa17 | 1.99966735 | 0.00253813 |
| 265 | Defa23 | 1.99966735 | 0.00253813 |
| 266 | Defa3 | 1.99966735 | 0.00253813 |
| 267 | Defa6 | 1.99966735 | 0.00253813 |
| 268 | Gm15284 | 1.99966735 | 0.00253813 |
| 269 | Nrk | 2.06047966 | 0.00140081 |
| 270 | Foxd3 | 2.06342637 | 0.00123636 |
| 271 | Defa-rs1 | 2.07524828 | 0.0181601 |
| 272 | Defa26 | 2.07524828 | 0.0181601 |
| 273 | Defa4 | 2.07524828 | 0.0181601 |
| 274 | Gm14851 | 2.07524828 | 0.0181601 |
| 275 | Defa-rs2 | 2.08612387 | 0.00187493 |
| 276 | Serpinc1 | 2.15871387 | 0.00322823 |
| 277 | Stx1b | 2.16395103 | 0.03609969 |
| 278 | Gm10104 | 2.18054384 | 0.00210236 |
| 279 | Ifnb1 | 2.18093962 | 0.00428191 |
| 280 | S100g | 2.1922448 | 0.00043847 |
| 281 | Mttp | 2.19935083 | 0.03538957 |
| 282 | Ceacam12 | 2.20490328 | 0.03880811 |
| 283 | Tuba4a | 2.22597517 | 0.00466546 |
| 284 | Bub1 | 2.27367662 | 0.01995372 |
| 285 | Klk1b11 | 2.31482964 | 0.00471781 |
| 286 | Fgb | 2.36843472 | 0.0062076 |
| 287 | Pgs1 | 2.53698101 | 0.00895029 |
| 288 | Cyp3a11 | 2.79948423 | 0.00271159 |
| 289 | Cyp3a16 | 2.79948423 | 0.00271159 |
| 290 | Reg3b | 2.86502188 | 0.00238112 |
| 291 | Reg3g | 3.06689373 | 0.0071675 |
| 292 | Fbxo3 | 3.40045449 | 0.04881924 |
