# Supplementary material for: Alcoholic fatty liver disease inhibited the co-expression of Fmo5 and PPARα to activate the NF-κB signaling pathway, thereby reducing liver injury via inducing gut microbiota disturbance
Source: J Exp Clin Cancer Res. 2021 Jan 7;40:18. doi: 10.1186/s13046-020-01782-w (PMC7788704; doi:10.1186/s13046-020-01782-w)
Supplement: Supplementary file 2 — Additional file 2. [file 13046_2020_1782_MOESM2_ESM.docx]

The supplementary information 2: DEGs in GSE40334 dataset

| ID | Genesymbol | logFC | P.Value |
| --- | --- | --- | --- |
| 1 | Cyp7a1 | -4.1859382 | 7.20E-05 |
| 2 | Sqle | -3.2910606 | 1.42E-06 |
| 3 | Tgfb1i4 | -3.1091789 | 1.77E-06 |
| 4 | Hsd3b5 | -3.0318387 | 0.03894818 |
| 5 | Socs2 | -2.6777506 | 0.00025238 |
| 6 | G0s2 | -2.4251931 | 8.51E-06 |
| 7 | Irs2 | -2.3675599 | 0.00204564 |
| 8 | Hsd3b4 | -2.2858191 | 2.20E-06 |
| 9 | Dct | -2.1904542 | 0.00032508 |
| 10 | Paqr9 | -2.1506721 | 0.00011316 |
| 11 | D0H4S114 | -2.0693313 | 0.00053235 |
| 12 | Rsb30 | -2.053949 | 3.19E-05 |
| 13 | Fgf21 | -2.0400894 | 0.00164934 |
| 14 | Cyp4a12 | -2.0326501 | 0.00377802 |
| 15 | Pte2a | -2.0056301 | 0.00517966 |
| 16 | Serpina12 | -1.9626659 | 0.0180812 |
| 17 | Arrdc3 | -1.9543065 | 0.00156666 |
| 18 | Mvd | -1.9103466 | 6.44E-05 |
| 19 | Dbp | -1.9046807 | 2.96E-06 |
| 20 | Kiss1 | -1.882695 | 0.00185893 |
| 21 | D130043K22Rik | -1.8562534 | 5.85E-06 |
| 22 | D830014E11Rik | -1.8172632 | 0.00178109 |
| 23 | Lamb3 | -1.808328 | 1.92E-05 |
| 24 | Fzd8 | -1.7232408 | 0.00021679 |
| 25 | Bcl6 | -1.7078984 | 0.00561373 |
| 26 | OTTMUSG00000000231 | -1.7003744 | 0.00015488 |
| 27 | Nudt7 | -1.6406953 | 0.00265465 |
| 28 | Gadd45a | -1.6305985 | 0.00254382 |
| 29 | Gps1 | -1.5987382 | 0.01919143 |
| 30 | Slc17a8 | -1.5849152 | 0.00193681 |
| 31 | Elovl3 | -1.5578515 | 0.01858909 |
| 32 | Id2 | -1.4815599 | 0.00433641 |
| 33 | Gm106 | -1.4783175 | 0.00101184 |
| 34 | Aqp4 | -1.4638794 | 0.00464377 |
| 35 | Isgf3g | -1.4067184 | 0.00066243 |
| 36 | 9130020G10Rik | -1.4037363 | 0.00166929 |
| 37 | Nr1d1 | -1.3764918 | 0.00075481 |
| 38 | Idb4 | -1.3592489 | 0.00302442 |
| 39 | C6 | -1.3422177 | 0.00142814 |
| 40 | 1700024D23Rik | -1.3159732 | 0.00707278 |
| 41 | Hsd17b9 | -1.3051514 | 0.00045989 |
| 42 | Abhd2 | -1.3015018 | 6.26E-05 |
| 43 | Treh | -1.2772791 | 0.00012036 |
| 44 | 2200001I15Rik | -1.2738932 | 9.17E-05 |
| 45 | Efna1 | -1.2684899 | 0.01830544 |
| 46 | Idb1 | -1.2451931 | 0.0045114 |
| 47 | Tmeff1 | -1.2359755 | 0.00029183 |
| 48 | 9130422G05Rik | -1.2286142 | 0.00075171 |
| 49 | Omd | -1.2226827 | 0.0026498 |
| 50 | MGC25972 | -1.2177615 | 0.02127434 |
| 51 | Idb3 | -1.2159884 | 0.00682947 |
| 52 | Pcsk4 | -1.2141094 | 0.00022257 |
| 53 | Sucnr1 | -1.2132947 | 7.53E-05 |
| 54 | Olfr97 | -1.2033966 | 0.00023818 |
| 55 | Cbx4 | -1.1879983 | 0.00039628 |
| 56 | Cyp7b1 | -1.1775162 | 0.00889605 |
| 57 | Dio1 | -1.1749611 | 0.00036117 |
| 58 | E430021N18Rik | -1.1749279 | 0.00097208 |
| 59 | Fmo3 | -1.169024 | 0.00822087 |
| 60 | Tff3 | -1.1676912 | 0.00099378 |
| 61 | Inhbe | -1.1645243 | 0.00023838 |
| 62 | EG624219 | -1.1630731 | 0.00191721 |
| 63 | Tenc1 | -1.1615524 | 0.0025893 |
| 64 | Bmf | -1.1556071 | 0.03606939 |
| 65 | Crat | -1.1523928 | 6.75E-05 |
| 66 | Junb | -1.1398959 | 0.02253823 |
| 67 | 1110001A05Rik | -1.1371156 | 0.0209464 |
| 68 | Cyp2u1 | -1.1246803 | 0.00145799 |
| 69 | Syt8 | -1.117901 | 0.00529555 |
| 70 | Wdfy2 | -1.1068514 | 0.00039012 |
| 71 | 2510004L01Rik | -1.1055932 | 0.00016075 |
| 72 | Srd5a1 | -1.0986629 | 0.00064873 |
| 73 | Foxa2 | -1.0920014 | 0.01475076 |
| 74 | Ppap2b | -1.0874733 | 0.00462194 |
| 75 | 4930481F22Rik | -1.0866659 | 4.91E-05 |
| 76 | 1700023E05Rik | -1.0856374 | 0.0006333 |
| 77 | Sca2 | -1.0795996 | 0.00709622 |
| 78 | Stard4 | -1.0735809 | 0.00534896 |
| 79 | Gnat1 | -1.0709141 | 0.01033315 |
| 80 | AI586015 | -1.0700487 | 0.00368585 |
| 81 | Trpv5 | -1.0571219 | 0.02239582 |
| 82 | Prlr | -1.0562481 | 0.01253767 |
| 83 | Crls1 | -1.0488944 | 0.00349127 |
| 84 | Ms4a10 | -1.0462158 | 0.00204833 |
| 85 | BC057371 | -1.0429631 | 0.00458082 |
| 86 | 6330404A07Rik | -1.0425339 | 0.0007232 |
| 87 | Has1 | -1.0388355 | 0.01098935 |
| 88 | 1200013B22Rik | -1.0330553 | 0.00146302 |
| 89 | Brdt | -1.0301746 | 0.00651031 |
| 90 | Adprhl1 | -1.0217873 | 0.00255344 |
| 91 | Atp9a | -1.0211071 | 0.00053706 |
| 92 | LOC620631 | -1.013476 | 0.02944671 |
| 93 | C1qtnf4 | -1.0058473 | 0.00376464 |
| 94 | Phlda1 | -1.0046661 | 0.03839728 |
| 95 | Hist2h2ab | -1.0045028 | 0.00047545 |
| 96 | Cebpa | -1.0033428 | 0.00182565 |
| 97 | Pscd2 | -1.0030739 | 0.00099585 |
| 98 | Olfr802 | -1.0009206 | 0.01781707 |
| 99 | Smpd3 | 1.00015822 | 0.00184972 |
| 100 | Gal3st1 | 1.00264223 | 0.00543849 |
| 101 | S100a9 | 1.00344129 | 0.02083001 |
| 102 | Cryl1 | 1.00362764 | 0.00032119 |
| 103 | S100a11 | 1.00526924 | 0.01779922 |
| 104 | Etnk2 | 1.00747409 | 0.01184344 |
| 105 | Ppp1r3b | 1.00794627 | 0.01101742 |
| 106 | Crym | 1.00824578 | 0.01214783 |
| 107 | Psmb7 | 1.0115848 | 0.03844537 |
| 108 | Mest | 1.01220348 | 0.00335871 |
| 109 | Hrsp12 | 1.01441197 | 0.00970583 |
| 110 | Cyp2c39 | 1.01832912 | 0.00907164 |
| 111 | Ctps | 1.01852726 | 0.00244265 |
| 112 | Lmyc1 | 1.02238917 | 0.00110876 |
| 113 | CRG-L1 | 1.02389054 | 0.00153997 |
| 114 | 9130409I23Rik | 1.02716129 | 0.00509918 |
| 115 | S100a8 | 1.02832306 | 0.04909084 |
| 116 | 2210413P12Rik | 1.02927375 | 0.00113992 |
| 117 | 9330177P20Rik | 1.03248671 | 0.01254877 |
| 118 | Msc | 1.03359816 | 0.00293152 |
| 119 | 4930542G03Rik | 1.03701658 | 0.00014295 |
| 120 | Akr1c12 | 1.04726597 | 0.01183521 |
| 121 | Trim2 | 1.05640919 | 0.03629658 |
| 122 | 5730538E15Rik | 1.05926778 | 0.00130337 |
| 123 | Map4k4 | 1.06146785 | 0.01241439 |
| 124 | Gypc | 1.06402209 | 0.00042461 |
| 125 | 1300018P11Rik | 1.07158414 | 0.00367904 |
| 126 | D430039N05Rik | 1.07158478 | 0.00945187 |
| 127 | Nfat5 | 1.07741972 | 0.00022868 |
| 128 | Htatip2 | 1.08026633 | 0.00026188 |
| 129 | Uhrf1 | 1.08079807 | 0.03411833 |
| 130 | Hbb-y | 1.08263734 | 0.00310897 |
| 131 | Il13ra2 | 1.08481016 | 0.0091459 |
| 132 | Epb4.1l1 | 1.08498563 | 0.04106526 |
| 133 | Lin7b | 1.08872091 | 0.00072967 |
| 134 | Akr1c19 | 1.08884493 | 0.0007064 |
| 135 | Gemin6 | 1.08894824 | 0.00188317 |
| 136 | Cdc2a | 1.09524571 | 0.00420641 |
| 137 | Ncoa6 | 1.1102619 | 0.0047742 |
| 138 | BC064033 | 1.11149077 | 0.0385192 |
| 139 | BC023882 | 1.11565358 | 0.00022724 |
| 140 | Tmem18 | 1.1183068 | 0.000564 |
| 141 | Gstt3 | 1.12414648 | 0.00024157 |
| 142 | Camsap1l1 | 1.13048832 | 0.0003296 |
| 143 | Saa2 | 1.13719883 | 0.00016612 |
| 144 | Npn3 | 1.13812902 | 0.00015679 |
| 145 | Tmc7 | 1.14458464 | 0.01205335 |
| 146 | Stmn1 | 1.14750618 | 0.0004699 |
| 147 | Thrsp | 1.15022527 | 0.02759346 |
| 148 | LOC329575 | 1.15445014 | 0.00364026 |
| 149 | Sparc | 1.16095413 | 0.00255719 |
| 150 | Ctsj | 1.166407 | 0.0008986 |
| 151 | Orm1 | 1.16864005 | 0.01051076 |
| 152 | Slc13a2 | 1.17361638 | 0.00916294 |
| 153 | Gabrb3 | 1.18253799 | 0.00106996 |
| 154 | 4833442J19Rik | 1.18505669 | 0.00040584 |
| 155 | 2310057H16Rik | 1.18756653 | 1.09E-05 |
| 156 | LOC232400 | 1.18912767 | 0.00236988 |
| 157 | 9030624L02Rik | 1.19996702 | 0.00082881 |
| 158 | Tubb2b | 1.20369192 | 0.00010472 |
| 159 | Shrm | 1.21898331 | 0.00099585 |
| 160 | 9330161F08Rik | 1.21927832 | 0.03375085 |
| 161 | Dsip1 | 1.22460095 | 0.00253496 |
| 162 | Adm | 1.23762013 | 0.00257037 |
| 163 | Mgst3 | 1.24006559 | 0.00261906 |
| 164 | Cd63 | 1.24056659 | 0.01609793 |
| 165 | Ptpn8 | 1.24130257 | 6.26E-05 |
| 166 | Nol3 | 1.24458922 | 0.01653387 |
| 167 | Pfn2 | 1.24630399 | 0.0089302 |
| 168 | Plcd3 | 1.24881917 | 0.00490708 |
| 169 | Tuba7 | 1.25257492 | 9.43E-05 |
| 170 | Per1 | 1.25852772 | 0.02754878 |
| 171 | Adam11 | 1.26245636 | 0.0005737 |
| 172 | 1810011O10Rik | 1.26656472 | 0.00153859 |
| 173 | Cyp39a1 | 1.27473875 | 0.0146302 |
| 174 | BC048546 | 1.27503943 | 0.00144176 |
| 175 | Gspt2 | 1.27773736 | 0.00100515 |
| 176 | Gstm4 | 1.2856961 | 0.00171711 |
| 177 | Fxyd6 | 1.29252804 | 0.01868469 |
| 178 | Ggtla1 | 1.31818752 | 0.01266887 |
| 179 | Col6a2 | 1.32840705 | 0.00026898 |
| 180 | Agxt2l1 | 1.33118256 | 0.0005472 |
| 181 | AU040576 | 1.34090115 | 0.00045481 |
| 182 | Nusap1 | 1.35895381 | 0.00379991 |
| 183 | Tuba8 | 1.36142593 | 6.03E-05 |
| 184 | Lgals1 | 1.3752406 | 2.13E-05 |
| 185 | Amn | 1.39553782 | 0.01234255 |
| 186 | Rgs16 | 1.40028994 | 0.02176738 |
| 187 | Esm1 | 1.40305724 | 0.00730515 |
| 188 | Kcnk1 | 1.41842044 | 0.01040843 |
| 189 | Mt1 | 1.42903298 | 0.00085842 |
| 190 | Garnl4 | 1.43247943 | 0.00932417 |
| 191 | Psat1 | 1.44857747 | 0.00266235 |
| 192 | BC063749 | 1.45496427 | 0.00018906 |
| 193 | Slc13a3 | 1.47240189 | 0.00156847 |
| 194 | Mlkl | 1.48352481 | 0.00050885 |
| 195 | Ctgf | 1.52714391 | 0.00576728 |
| 196 | Ngfa | 1.53220386 | 7.89E-06 |
| 197 | Tsc22d3 | 1.53666708 | 0.001621 |
| 198 | Rhbg | 1.56462248 | 9.36E-06 |
| 199 | S3-12 | 1.56746523 | 8.55E-05 |
| 200 | Sdcbp2 | 1.5954061 | 0.00023723 |
| 201 | 2210402C18Rik | 1.60220651 | 0.00105782 |
| 202 | 1810008K03Rik | 1.6073539 | 0.00703436 |
| 203 | Orm2 | 1.63146032 | 0.00809226 |
| 204 | Ccnb1 | 1.65231396 | 0.00853645 |
| 205 | Slc39a4 | 1.65995177 | 0.00078602 |
| 206 | AI428936 | 1.67611114 | 0.01056538 |
| 207 | Gstm2 | 1.68799892 | 0.00034194 |
| 208 | Gck | 1.69138449 | 1.47E-05 |
| 209 | Fmo5 | 1.69340965 | 8.18E-05 |
| 210 | Tsku | 1.7143691 | 8.42E-06 |
| 211 | Mmp12 | 1.72751491 | 0.04754903 |
| 212 | Pin1 | 1.73582893 | 0.00025023 |
| 213 | AI595366 | 1.79961191 | 1.82E-05 |
| 214 | Mafb | 1.84457283 | 0.00275215 |
| 215 | Nudt11 | 1.86851488 | 2.81E-05 |
| 216 | Prc1 | 1.87159188 | 0.00022011 |
| 217 | Cdkn1a | 1.89188625 | 0.00017024 |
| 218 | Dyrk3 | 1.90183139 | 6.29E-05 |
| 219 | Lcn2 | 1.93054126 | 0.00014961 |
| 220 | Gsta4 | 1.93513645 | 2.72E-06 |
| 221 | Dfna5h | 1.954425 | 5.62E-06 |
| 222 | Cdc20 | 1.95540749 | 6.27E-05 |
| 223 | Dusp4 | 1.96285811 | 4.95E-05 |
| 224 | Rec8L1 | 1.99365428 | 3.35E-05 |
| 225 | Gstm1 | 2.01793884 | 8.02E-07 |
| 226 | Cyp2c55 | 2.04995902 | 0.0014593 |
| 227 | Raet1a | 2.0681243 | 3.10E-06 |
| 228 | Raet1b | 2.08065676 | 5.56E-05 |
| 229 | Klk8 | 2.10204574 | 0.02046529 |
| 230 | Cyp3a11 | 2.12727153 | 1.07E-05 |
| 231 | Nudt10 | 2.22766169 | 0.00069595 |
| 232 | Mt2 | 2.25841978 | 0.00652506 |
| 233 | 4922503N01Rik | 2.29852063 | 9.37E-07 |
| 234 | Cyp2a5 | 2.47396842 | 0.00031867 |
| 235 | Ddit4 | 2.70081037 | 0.0004083 |
| 236 | Lpin1 | 2.76101317 | 0.00015733 |
| 237 | 1810023F06Rik | 2.88041477 | 1.70E-06 |
| 238 | Cbr3 | 2.99542981 | 2.29E-06 |
| 239 | Cyp26a1 | 3.21397088 | 4.74E-05 |
| 240 | Gsta2 | 3.42324348 | 7.86E-06 |
| 241 | Gstm3 | 4.04932495 | 4.12E-07 |
| 242 | EG243881 | 4.92178849 | 7.96E-07 |
| 243 | Cyp2b9 | 5.20036524 | 5.54E-08 |
| 244 | Gsta1 | 5.71627807 | 1.13E-07 |
| 245 | Cyp2b13 | 6.3173749 | 1.04E-07 |
| 246 | Cyp2b20 | 7.36285518 | 1.21E-10 |
